# Tomato *POLLEN DEFICIENT 2* encodes a G-type lectin receptor kinase required for viable pollen grain formation

**DOI:** 10.1093/jxb/erac419

**Published:** 2022-10-19

**Authors:** Rosa Micol-Ponce, Manuel García-Alcázar, Ricardo Lebrón, Carmen Capel, Benito Pineda, Begoña García-Sogo, Juan de Dios Alché, Ana Ortiz-Atienza, Sandra Bretones, Fernando Juan Yuste-Lisbona, Vicente Moreno, Juan Capel, Rafael Lozano

**Affiliations:** Centro de Investigación en Biotecnología Agroalimentaria (CIAIMBITAL), Universidad de Almería, 04120 Almería, Spain; Centro de Investigación en Biotecnología Agroalimentaria (CIAIMBITAL), Universidad de Almería, 04120 Almería, Spain; Centro de Investigación en Biotecnología Agroalimentaria (CIAIMBITAL), Universidad de Almería, 04120 Almería, Spain; Centro de Investigación en Biotecnología Agroalimentaria (CIAIMBITAL), Universidad de Almería, 04120 Almería, Spain; Instituto de Biología Molecular y Celular de Plantas (UPV-CSIC), Universidad Politécnica de Valencia, 46011 Valencia, Spain; Instituto de Biología Molecular y Celular de Plantas (UPV-CSIC), Universidad Politécnica de Valencia, 46011 Valencia, Spain; Departamento de Bioquímica, Biología Celular y Molecular de Plantas, Estación Experimental del Zaidín-CSIC, 18008 Granada, Spain; Centro de Investigación en Biotecnología Agroalimentaria (CIAIMBITAL), Universidad de Almería, 04120 Almería, Spain; Centro de Investigación en Biotecnología Agroalimentaria (CIAIMBITAL), Universidad de Almería, 04120 Almería, Spain; Centro de Investigación en Biotecnología Agroalimentaria (CIAIMBITAL), Universidad de Almería, 04120 Almería, Spain; Instituto de Biología Molecular y Celular de Plantas (UPV-CSIC), Universidad Politécnica de Valencia, 46011 Valencia, Spain; Centro de Investigación en Biotecnología Agroalimentaria (CIAIMBITAL), Universidad de Almería, 04120 Almería, Spain; Centro de Investigación en Biotecnología Agroalimentaria (CIAIMBITAL), Universidad de Almería, 04120 Almería, Spain; University of Nottingham, UK

**Keywords:** Male sterility, mapping-by-sequencing, microgametogenesis, microsporogenesis, parthenocarpic fruits, pollen formation, receptor kinases, RNA interference, tapetum development, tomato

## Abstract

Pollen development is a crucial biological process indispensable for seed set in flowering plants and for successful crop breeding. However, little is known about the molecular mechanisms regulating pollen development in crop species. This study reports a novel male-sterile tomato mutant, *pollen deficient 2* (*pod2*), characterized by the production of non-viable pollen grains and resulting in the development of small parthenocarpic fruits. A combined strategy of mapping-by-sequencing and RNA interference-mediated gene silencing was used to prove that the *pod2* phenotype is caused by the loss of *Solanum lycopersicum* G-type lectin receptor kinase II.9 (SlG-LecRK-II.9) activity. *In situ* hybridization of floral buds showed that *POD2/SlG-LecRK-II.9* is specifically expressed in tapetal cells and microspores at the late tetrad stage. Accordingly, abnormalities in meiosis and tapetum programmed cell death in *pod2* occurred during microsporogenesis, resulting in the formation of four dysfunctional microspores leading to an aberrant microgametogenesis process. RNA-seq analyses supported the existence of alterations at the final stage of microsporogenesis, since we found tomato deregulated genes whose counterparts in Arabidopsis are essential for the normal progression of male meiosis and cytokinesis. Collectively, our results revealed the essential role of *POD2/SlG-LecRK-II.9* in regulating tomato pollen development.

## Teaser

*POD2*encodes a tomato G-type lectin receptor kinase vital for functional pollen formation. The loss of *POD2*function impairs meiosis and tapetum degradation, resulting in male sterility and parthenocarpy.

## Introduction

Growing enough food to sustain the increasing human population is one of the most important challenges today. Indeed, it has been estimated that food production should be increased by 70% in 2050 to meet the global demand (Fan *et al*., 2014). As a result, over the past decades many resources have been devoted to investigating how to improve plant yield. One of the most widely accepted solutions is the pursuit of plant heterosis or hybrid vigor, which can increase the biomass by 3.5–15% (Longin *et al.*, 2012). To achieve plant heterosis, different approaches have been taken to foster cross-pollination in place of self-fertilization. Most research to date has focused on methods to promote cross-pollination rather than on a better understanding of plant fertility and pollen development regulation.

The development of the male gametophyte, or pollen grain, takes place within the anther and entails a plethora of biological processes that require coordinated activity of both sporophytic and gametophytic cell types, leading to the release of functional pollen and plant fertilization (Fernández-Gómez *et al.*, 2015). Pollen development comprises two consecutive and differentiated developmental phases, microsporogenesis and microgametogenesis. Throughout microsporogenesis, diploid archesporial cells differentiate into meiocytes or microspore mother cells (MMCs) enclosed by the sporophytic anther wall layers. The most conspicuous anther cell layer is the tapetum, which undergoes developmentally regulated programmed cell death to function as a nurse tissue essential for proper pollen formation; indeed, mutations that disrupt this developmental program promote aborted microgametogenesis causing male sterility (Hafidh *et al.*, 2016). The MMCs undergo meiotic division to produce tetrads of haploid microspores bound by their cell walls, which are largely composed of the polysaccharide callose. Microsporogenesis is completed by the digestion of the tetrad cell wall, leading to the release of single-celled haploid microspores, which develop into mature pollen grains during the microgametogenesis phase. An outstanding milestone in microsporogenesis is the proper segregation of the products of male meiosis into single-celled haploid microspores, as indicated by studies in Arabidopsis mutants such as *tetraspore* and *stud*. These mutations generate a single abnormal polyploid microspore instead of four haploid tetrads due to the failure of male meiotic cytokinesis, affecting pollen functionality (Yang *et al.*, 2003). Once a haploid microspore is formed, it undergoes an asymmetrical mitotic division giving rise to an early bicellular pollen grain formed of a large vegetative cell and a small generative cell, which, in turn, forms two sperm cells or male gametes through mitotic division. Finally, pollen development concludes with the release of mature pollen grains from the anthers at flower anthesis (Hafidh *et al.*, 2016).

Fruit set depends on the successful completion of pollination and fertilization, so that the growth of the ovary is inhibited by negative regulatory factors stemming from anther–ovary communication in the absence of fertilization (de Jong *et al.*, 2009). The flower-to-fruit transition is a very sensitive process due to its vulnerability to endogenous and exogenous factors. Unfavorable environmental conditions, such as excessively high or low temperatures, can disrupt the reproductive process, resulting in flower or fruit abortion (Bita *et al.*, 2011). In parthenocarpy, ovary growth is not inhibited, resulting in a seedless fruit through a flower-to-fruit transition process that is less dependent on environmental conditions (Gorguet *et al.*, 2005). In male-sterile mutants, parthenocarpy is successfully induced, which makes them one of the most useful tools for investigating the mechanisms underlying flower-to-fruit transition. In Arabidopsis, anther development has been further studied, and mutations affecting genes involved in each stage of male gametophyte development have been described (Twell, 2010). Tomato pollen development is similar to that of Arabidopsis and rice (Brukhin *et al.*, 2003; Wilson and Zhang, 2009), and while several dozens of mutants exhibiting impaired pollen development were isolated many years ago, only a few causal genes have been identified. For instance, around 50 tomato male-sterile mutants altered in different pollen developmental stages were described by Rick and Butler (1956), but only a few causal genes have been described thus far. A single nucleotide mutation in a gene encoding a polygalacturonase is responsible for the *positional sterility-2* phenotype conferring non-dehiscent anthers (Gorguet *et al.*, 2009), whereas an insertion of a retrotransposable DNA fragment in the promoter region of a bHLH transcription factor gives rise to dysfunctional meiosis and abnormal tapetum development in the *male sterile10*^*35*^ (*ms10*^*35*^) mutant (Jeong *et al.*, 2014; Jung *et al.*, 2020). Another bHLH transcription factor and the B-class MADS-box *TM6* gene have been also proposed as candidate genes for *ms32* and *ms15*^*26*^ phenotypes (Cao *et al.*, 2019; Liu *et al.*, 2019). Furthermore, the tomato *pollen deficient 1* mutant has been described as a recessive null allele of the *SIMED18* gene encoding subunit 18 of the well-conserved eukaryotic Mediator complex (Pérez-Martín *et al.*, 2018). This mutation causes a reduction in the amount of viable pollen as a consequence of a delayed tapetum degeneration.

Receptor kinases (RKs) are transmembrane proteins with an extracellular region (ectodomain) and an intracellular kinase domain, which play critical roles in perceiving environmental signals and/or developmental cues to regulate plant reproductive development (Cai and Zhang, 2018; Kim *et al.*, 2021). Indeed, RKs are essential for pollen biogenesis and maturation. During these processes, they function and exhibit specific expression profiles that are conserved across species (Julca *et al.*, 2021), including tomato, where the characterization of the *small parthenocarpic fruit and flower* mutant has led to the identification of a novel RK protein required for male fertility (Takei *et al.*, 2019). RKs have been classified into two groups according to their ectodomain: a leucine-rich repeat domain (LRR-RKs) or a lectin domain (LecRKs). LecRKs are further subdivided into three types, based on the lectin domain that they harbor: the G- (*Galanthus nivalis* agglutinin), L- (legume-like), and C- (calcium-dependent)-type lectin domain. G-type LecRKs (G-LecRKs) contain an ectodomain that resembles the *G. nivalis* agglutinin mannose-binding motif (Lannoo and van Damme, 2014). A total of 38 and 73 G-LecRKs have been identified to date in Arabidopsis and tomato, respectively, denoting an expansion of these G-LecRKs in tomato, possibly due to combinations of tandem and whole-genome duplications (Teixeira *et al.*, 2018).

Here, we describe the tomato *pollen deficient 2* (*pod2*) mutant, which was isolated in a genetic screen for reproductive impairment using a collection of tomato enhancer trap lines in a cv. Moneymaker background (Pérez-Martín *et al.*, 2017). The *pod2* mutant is characterized by the production of abnormal pollen grains, which causes the formation of small parthenocarpic fruits. Following a combined strategy of mapping-by-sequencing and RNA interference (RNAi)-mediated gene silencing, we found that *POD2* encodes the *Solanum lycopersicum* G-type lectin receptor kinase II.9 (SlG-LecRK-II.9), thus revealing for the first time its essential role in the correct development and maturation of pollen grains in tomato.

## Materials and methods

### Plant material and growth conditions

The *pod2* mutant was isolated from a T-DNA insertional collection generated in the cv. Moneymaker genetic background (Pérez-Martín *et al.*, 2017). Because the *pod2* homozygous plants were not able to produce viable seeds, the *POD2*/*pod2* heterozygous plants were self-pollinated to preserve the *pod2* mutant line. Plants were cultivated in soil with the regular addition of fertilizers using standard practices under greenhouse conditions.

### Phenotypic characterization

To determine whether there were any differences in phenotypic characteristics of agronomic relevance between the *pod2* mutant and the wild-type cv. Moneymaker, the fruit set percentage, and the total fruit yield at the second and third inflorescences were assessed in 15 plants of each genotype. In addition, five fruits per plant were selected and the weight, length, diameter, and number of seeds of each fruit were evaluated. In all cases, plants and fruits were chosen randomly. Phenotypic characteristics with *P-*values ≤0.01 by Student’s *t*-test were considered to be significantly different between the two genotypes.

### Evaluation of stigma receptivity and pollen viability

Stigma receptivity and *in vivo* pollen viability were evaluated by aniline blue staining and fluorescence microscopy as described by Pérez-Martín *et al.* (2018). For this experiment, pre-anthesis flowers were emasculated and kept enclosed in a cap. At anthesis, these flowers were hand-pollinated, and then collected 3 days after pollination and sectioned. Carpels and pistils from hand-pollinated flowers were then fixed for at least 24 h in FAE [formaldehyde:acetic acid:70% ethanol 1:2:17 (v:v:v)]. Tissues were rinsed in tap water overnight at 4 °C, subsequently softened with 0.8 N NaOH for 6 h, and rinsed again in tap water as previously. Pollen tubes were stained for 2 h in the dark with 0.1% aniline blue (w/v) in 0.1 N K_3_PO_4_. Fluorescence of stained pollen tubes was visualized with an Optiphot-2 (Nikon) optical microscope equipped with an HB-10101AF Mercury Lamp (Nikon).

For testing pollen viability, fresh pollen grains were stained using 2,3,5-triphenyltetrazolium chloride (TTC). Fresh pollen grains were harvested from anthers with a needle and dropped on to a slide. Then, a solution of 0.5% TTC in 0.5 M sucrose was added, and the mixture was quickly enclosed with a slide cover. Samples were incubated in the dark at 50 °C in a humidity chamber for 2 h. Viable grains turned red while non-viable grains remained colorless. A Nikon Optiphot-2 bright-field microscope equipped with a Nikon digital camera was used to observe the pollen grains.

### Pollen development analysis and microscopy

A comparative study of pollen ontogeny was conducted by using light microscopy (LM) and transmission electron microscopy (TEM) as described by Pérez-Martín *et al.* (2018) and Jimenez-Lopez *et al.* (2016), respectively. Anther sections 7 μm thick were cut and a mixture of toluidine blue and methylene blue was used for staining. A Nikon Eclipse Ti-U microscope was employed to visualize anther sections by LM. For TEM, 80 nm ultrathin sections were mounted on formvar-coated 200-mesh nickel grids, and contrasted with uranyl acetate substitute and lead citrate. Observations were made with a Jeol JEM1011 transmission electron microscope at 80 kV. For scanning electron microscopy (SEM) analyses, flowers from each genotype were fixed in a mixture of FAEG (10% formaldehyde, 5% acetic acid, 50% absolute ethanol, and 0.72% glutaraldehyde). Samples were washed and dehydrated in ethanol (70–100%). Critical point drying with liquid CO_2_ was carried out after sample dehydration in a Bal Tec CPD 030 critical point drier. Samples were coated with gold by a Bal Tec SCD005 sputter coater and examined using a Hitachi S-3500N scanning electron microscope at 10 kV.

### Molecular identification of the *pod2* mutation

Plant DNAzol Reagent (Invitrogen Life Technologies, San Diego, CA, USA) was used to isolate genomic DNA from 100 mg of young leaves, following the instructions of the manufacturer. Primers specific to the T-DNA transformation vector pD991 (Pérez-Martín *et al.*, 2017) were used to detect T-DNA fragments in a T_1_ offspring. PCR assays showed that the T-DNA insertion did not cosegregate with the *pod2* phenotype. To identify the gene causing the *pod2* phenotype, a mutant plant with no T-DNA insertion was crossed to the LA1589 accession of the wild tomato *Solanum pimpinellifolium*. An F_2_ mapping population was generated by self-fertilizing the F_1_ plants. Mapping-by-sequencing was performed according to Yuste-Lisbona *et al.* (2021). Briefly, equal amounts of DNA from 25 randomly selected phenotypically wild type and 16 phenotypically mutant plants were pooled to construct wild-type and mutant pools, respectively. DNA pools were sequenced on the Illumina HiSeq2000 platform (Illumina, Inc., San Diego, CA, USA), which produced paired-end reads of 100 bp. The obtained sequences have been deposited at the Sequence Read Archive database at the National Center for Biotechnology Information under BioProject accession number PRJNA760263. The paired-end reads were aligned to the tomato genome reference sequence (ITAG4.0) using Bowtie2 with the default parameters (Langmead and Salzberg, 2012) and the duplicated reads were removed by MarkDuplicates, one of the Picard tools (http://broadinstitute.github.io/picard/). The HaplotypeCaller tool from GATK (https://gatk.broadinstitute.org/) was applied for variant calling analysis. Next, biallelic variants with a minimum depth of 10 reads per sample were filtered with BCFtools from the SAMtools package (http://www.htslib.org/). The allele frequency ratio for biallelic variants was calculated as non-reference allele counts divided by total allele counts. A sliding window and step size of 1000 and 100 variants, respectively, were used to calculate the average allele frequency per chromosome using a custom script in the R environment for statistical computing. Finally, the average allele frequencies along each chromosome were plotted with the aim of identifying the chromosomal region where the *pod2* mutation is located. The identified variants encompassing the candidate genomic region to host the *pod2* mutation were filtered based on two criteria: (i) the alternative allele in wild-type and mutant pool variants must be in a heterozygous (0/1) and homozygous (1/1) state, respectively; and (ii) alternative alleles must be unique variants that have not been previously reported in the sequenced tomato genomes (Lin *et al.*, 2014; The 100 Tomato Genome Sequencing Consortium, 2014).

The *POD2* locus was genotyped with a dCAPS marker using the POD2-F1 and POD2-R1 primers (Supplementary Table S1). A 96 bp fragment of the *Solyc04g015460* gene was amplified by PCR and digested with *Cla*I endonuclease (New England BioLabs, Ipswich, MA, USA). The mutant allele lacks the *Cla*I recognition site, whereas for the wild-type allele the 96 bp fragment was cleaved into 72 and 24 bp fragments. PCR amplification was carried out in a volume of 30 μl using 1 U of BIOTAQ^TM^ DNA Polymerase (Bioline, London, UK), 1× Taq buffer, 2.5 mM MgCl_2_, 0.25 mM dNTPs, 50 ng of each primer, and 10 ng of total DNA. Thermal cycling conditions used for DNA amplification were: 94 °C for 5 min, followed by five cycles at 94 °C for 15 s, 55 °C for 15 s, and 72 °C for 30 s, then 30 cycles at 94 °C for 15 s, 60 °C for 15 s, and 72 °C for 30 s, and a final extension step of 2 min at 72 °C. A PCR aliquot (10 µl) of each sample was digested for 2 h at 37 °C in 20 µl total volume with 1 U of *Cla*I endonuclease. The digestion products were electrophoresed in 2% agarose gels in 1× SB buffer (200 mM NaOH, 750 mM boric acid, pH 8.3) and stained with GelRed Nucleic Acid Stain (Biotium, Fremont, CA, USA).

### RNA preparation and gene expression analysis

TRIzol reagent (Invitrogen Life Technologies, San Diego, CA, USA) was employed to extract total RNA following the manufacturer’s instructions. A DNA-free^TM^ kit (Ambion, Austin, TX, USA) was used to remove genomic DNA contamination. The M-MuLV reverse transcriptase (Invitrogen Life Technologies, San Diego, CA, USA) and a mixture of random hexamer and 18-mer oligo(dT) primers were used to synthesize cDNA.

Expression analyses were performed on a 7300 Real-Time PCR System (Applied Biosystems, Foster City, CA, USA), using the SYBR Green PCR Master Mix kit and a specific primer pair for each gene analyzed (Supplementary Table S1). The ΔΔCt method was applied to calculate relative gene expression levels, using the housekeeping gene *UBIQUITINE3* as a reference. Significant differences among gene expression levels were determined by Fisher’s least significant difference test, using the Statgraphics Centurion XVI software package.

### *In situ* hybridization

Tissue preparation, sectioning, and transcript detection for *in situ* hybridization experiments were performed as described by Pérez-Martín *et al.* (2018). Briefly, to obtain sense (negative control) and antisense *POD2* RNA probes, a 218 bp fragment of the *Solyc04g015460* gene was PCR amplified from wild-type cDNA, using primers listed in Supplementary Table S1. The resulting PCR product was cloned into the pGEM^®^-T Easy Vector (Promega, Madison, WI, USA). After plasmid linearization, the DIG RNA labelling mix (Roche Applied Science, Indianapolis, IN, USA) and T7 or SP6 polymerases were used for *in vitro* transcription of sense and antisense probes, respectively.

### Generation of RNAi transgenic plants

An RNAi transgene was constructed to silence the *POD2* gene. A specific fragment of 389 bp from the *POD2* cDNA was obtained by PCR amplification using the primers RNAi_POD2_F (tctagactcgagACTTCCCATTATTCGCGTTG), to introduce an *Xba*I (T/CTAGA) and a *Xho*I (C/TCGAG) restriction site, and RNAi_POD2_R (atcgatggtaccTGGACCTGAAACTTGCTGTG), to introduce a *Cla*I (AT/CGAT) and a *Kpn*I (GGTAC/C) restriction site (Supplementary Table S1). The 389 bp PCR product was cloned into the pGEM^®^-T Easy Vector (Promega, Madison, WI, USA) and subsequently liberated twice by digestion: initially by *Xho*I and *Kpn*I digestion, and then by *Xba*I and *Cla*I digestion. The liberated fragments were cloned as inverted repeats into the pKannibal vector, which was later digested with *Not*I. The resulting fragment with the inverted 389 bp fragments of *POD2* separated by intronic sequences was finally cloned into the binary pART27 vector and electroporated into *Agrobacterium tumefaciens* strain LBA4404. Genetic transformation was conducted following the procedure previously described (Ellul *et al.*, 2003). Flow cytometry was used to evaluate the ploidy level of transgenic plants according to the methodology previously described (Pérez-Martín *et al.*, 2017). For further phenotypic and expression analyses, diploid transgenic lines were selected.

### Transcriptomic analysis

For gene expression analyses and comparisons, three biological replicates from flowers at three different stages related to pollen development: tetrad (floral buds of 3–4 mm), microspore (flowers of 5–6 mm) and young pollen (flowers of 7–8 mm) were sequenced for both wild-type and *pod2* mutant plants. TRIzol reagent (Invitrogen Life Technologies, San Diego, CA, USA) was used to isolate total RNA following the manufacturer’s instructions. A DNA-free^TM^ kit (Ambion, Austin, TX, USA) was employed to remove genomic DNA contamination. The Illumina TruSeq RNA protocol was followed to prepare sequencing libraries. The Illumina HiSeq2000 platform (Illumina, Inc., San Diego, CA, USA) was used to generate paired-end reads of 150 bp. The Illumina short reads were submitted to the Sequence Read Archive database at the National Center for Biotechnology Information under BioProject accession number PRJNA760265.

Sequencing reads were mapped against the tomato reference genome (ITAG4.0) using Tophat (Kim *et al.*, 2013) software with the following arguments: *--read-gap-length 12 --read-edit-dist 20 --read-mismatches 12 --segment-mismatches 3 --splice-mismatches 1 --no-coverage-search --read-realign-edit-dist 0 -g 1 --max-insertion-length 12 --max-deletion-length 12*. Transcript Per Million (TPM) normalization was applied to normalize read counts for gene/sample hierarchical (bi)clustering, heatmap representations, and sample comparisons. The consistency among RNA-seq replicates was checked using the top 5000 genes with the highest cumulative expression in all samples, which were selected for hierarchical biclustering analysis based on Z-scores of expression values as TPM. Two replicates (denoted Mc_mut3 and YP_mut3 in Supplementary Fig. S1) showed a clustering behavior that differed from the other two corresponding replicates, and were discarded for further analyses. The ComplexHeatmap R package (Gu *et al.*, 2016) was employed to obtain hierarchical biclustering and heatmaps using the complete cluster method with Euclidean distance for both genes and samples.

A differential expression analysis was conducted using the Wald test implemented in DESeq2 (Love *et al.*, 2014) to compare VST-normalized expression values of mutants versus wild type for each developmental stage. All genes with a false discovery rate (FDR) adjusted *P*-value ≤0.05 were defined as differentially expressed genes and classified as up-regulated or down-regulated according to their positive or negative log_2_ fold change values, respectively. Gene Ontology (GO) term enrichment analyses for the Biological Process category were conducted using EXPath2 (Tseng *et al.*, 2020) for each set of differentially expressed genes. All GO terms with an FDR adjusted *P*-value ≤0.05 were selected.

## Results

### The *pod2* mutant exhibits small parthenocarpic fruits

**Fig. 1.**
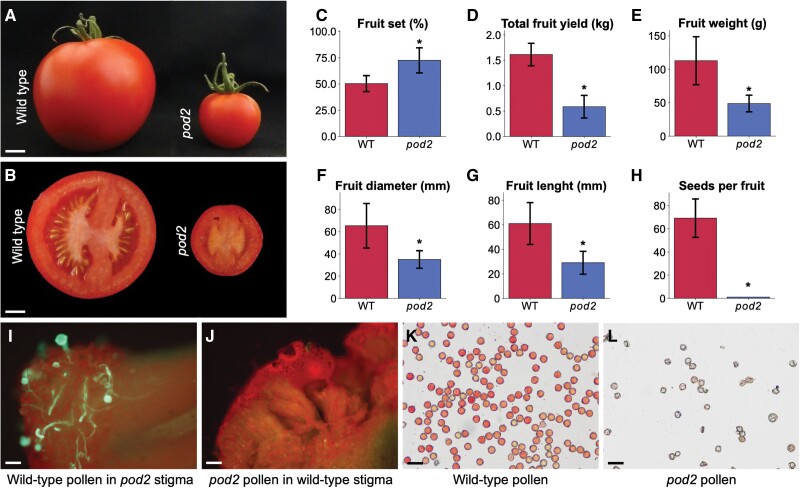
Phenotypic characterization of the *pod2* mutant. (A, B) Side view (A) and cross section (B) of wild-type and *pod2* fruits. (C–H) Comparison of yield- and fruit-related traits in wild-type and *pod2* plants: fruit set (C), total fruit yield (D), fruit weight (E), fruit diameter (F), fruit length (G), and seeds per fruit (H). Data are presented as means ±SD. Asterisks indicate significant differences between *pod2* and wild-type plants (*P≤0.001; Student’s *t*-test). (I, J) Hand-pollination of wild-type and *pod2* stigmas with *pod2* and wild-type pollen, respectively. (K, L) Pollen of wild-type (K) and *pod2* (L) plants stained with TTC. Scale bars=1 cm (A, B), 100 µm (I, J), and 50 µm (K, L).

The *pod2* mutant, which was isolated in a genetic screen for tomato reproductive impairment, was classified under the ‘fruit morphology and parthenocarpy’ phenotypic category (Pérez-Martín *et al.*, 2017) due to its small parthenocarpic fruits (Fig. 1A, B). Although the percentage of fruit set was significantly higher in *pod2* plants than in wild-type plants (Fig. 1C), the total fruit yield was significantly lower (Fig. 1D). Quantification of several parameters of *pod2* fruits showed that their weight, diameter, and length were significantly lower than those of wild-type fruits (Fig. 1E–G). Because the fruits of *pod2* were parthenocarpic, only one seed was found in a total of 75 fruits collected from 15 mutant plants (Fig. 1H).

As parthenocarpy may arise from the absence of pollen, inability of fertilization, or embryo abortion (Srivastava and Handa, 2005), we first focused the characterization of the *pod2* mutant on floral and pollen development. No differences in the floral organ morphology of wild-type and *pod2* plants were detected at the floral bud and mature flower stages. Epidermal stamen cells were observed by SEM, which revealed no alterations in the morphology or identity of *pod2* cells (Supplementary Fig. S2). To determine whether the *pod2* mutation impairs stigma or pollen development, we examined *in vivo* pollen germination and pollen tube elongation. We used the callose-specific stain aniline blue to easily visualize pollen tubes. Aniline blue staining showed that wild-type pollen grains germinated and developed normal pollen tubes on the stigmas of *pod2* flowers (Fig. 1I); by contrast, *pod2* pollen grains were unable to develop pollen tubes on wild-type stigmas (Fig. 1J; hand-pollination control test results are shown in Supplementary Fig. S3). Furthermore, strong evidence of defective pollen development in *pod2* plants was found upon analyzing the viability of their pollen grains by TTC staining (Fig. 1K, L). Compared with wild-type plants, the amount of pollen grains was ~83% lower in the anthers of *pod2* plants, and the *pod2* grains were poorly stained with TTC, indicating that they had very weak or no viability, whereas pollen grains of wild-type plants were strongly stained (Fig. 1K, L). Additionally, when *pod2* flowers were fertilized with wild-type pollen, they developed into fruits with viable seeds, indicating that female gametophyte development is not affected by the *pod2* mutation. These findings led us to conclude that *pod2* is a male-sterile mutant.

### Pollen development is impaired in *pod2* plants

**Fig. 2.**
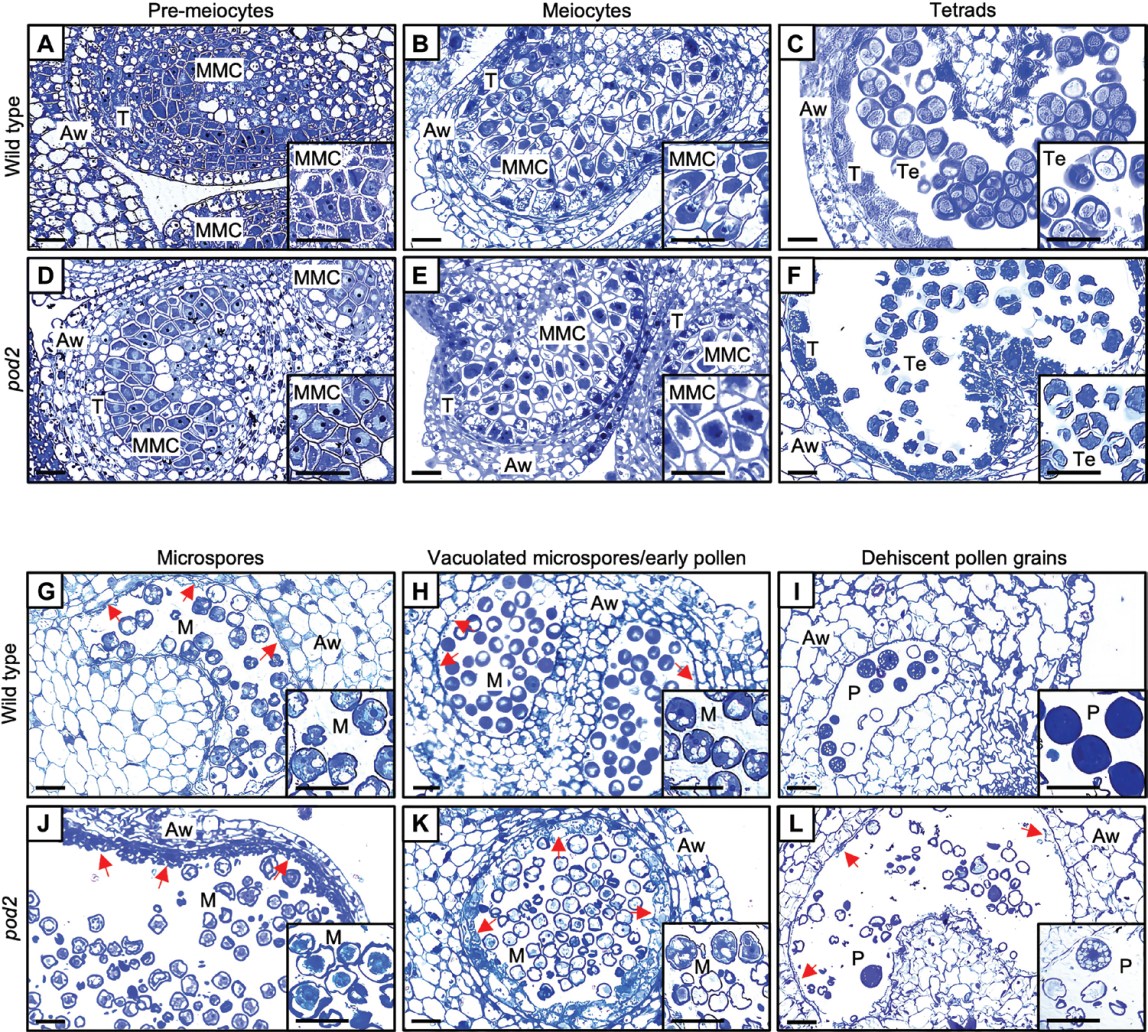
Histological characterization of anther development in wild-type and *pod2* flowers. Cross sections of anthers at pre-meiotic (A, D), meiotic (B, E), tetrad (C, F), microspore (G, J), mitotic (H, K), and dehiscence (I, L) stages. Aw, anther wall; M, microspores; MMC, microspore mother cells; P, pollen grains; T, tapetum; Te, tetrads. Red arrows indicate tapetum tissue. Scale bars=50 μm (main images) and 25 μm (inset images).

Pollen grain development takes place in anthers, where meiocytes undergo meiosis to form haploid microspores initially associated in a tetrad. Microspores are then released from the tetrad and undergo mitotic divisions to produce mature pollen (Hafidh *et al.*, 2016). To determine which stage of pollen development is affected by the *pod2* mutation, we stained sections of anthers of both wild-type and mutant plants at different stages of anther development with toluidine blue for LM analysis (Fig. 2). In the stages of pollen development prior to the tetrad (pre-meiocytes and meiocytes), we observed no differences between wild type and *pod2* (Fig. 2A, B, D, E). Morphological abnormalities were first noticed in *pod2* at the final stage of microsporogenesis, shortly before haploid microspores were released from the callose envelope. Thus, *pod2* MMCs were found to divide normally into tetrads and gave rise to four microspores (Fig. 2C, F), indicating that meiotic division is not affected. However, the microspores and callose envelope of the *pod2* tetrads showed an irregular surface and a deformed appearance. Furthermore, large morphological differences were identified between wild type and *pod2* at the microgametogenesis phase (Fig. 2G–L). Wild-type microspores became vacuolated and showed a polarized nucleus (Fig. 2G, H), whereas *pod2* microspores stained poorly with toluidine blue and showed an irregular shape with a barely recognizable nucleus, starting to demonstrate degeneration (Fig. 2J, K). Finally, at the dehiscence phase (Fig. 2I, L), the presence of empty pollen grains, which displayed evident symptoms of degeneration, was observed in *pod2*. Among the sporophytic anther cell layers, the epidermis, endothecium, and middle layer developed normally without any obvious defects, whereas a thicker tapetum was observed in the *pod2* mutant anther at the microspore and young pollen stages (Fig. 2J, K) in comparison with the wild type (Fig. 2G, H). Furthermore, a complete degeneration of the tapetum was not achieved in *pod2* mutant anthers, as a tiny tissue layer remained visible at the mature pollen stage while in wild-type anthers this tissue was completely resorbed to provide nutritional compounds for the developing pollen grains (Fig. 2I, L). To further analyze the alterations observed in the tapetal thickness, a morphometric analysis was performed during pollen formation, which revealed that, in agreement with the histological observations, the tapetum layer of *pod2* was significantly thicker than the wild-type tapetum at the vacuolated microspore and young pollen stages (Supplementary Fig. S4). Taken together, these results indicate that the *pod2* mutation affects both gametophytic and sporophytic tissues.

**Fig. 3.**
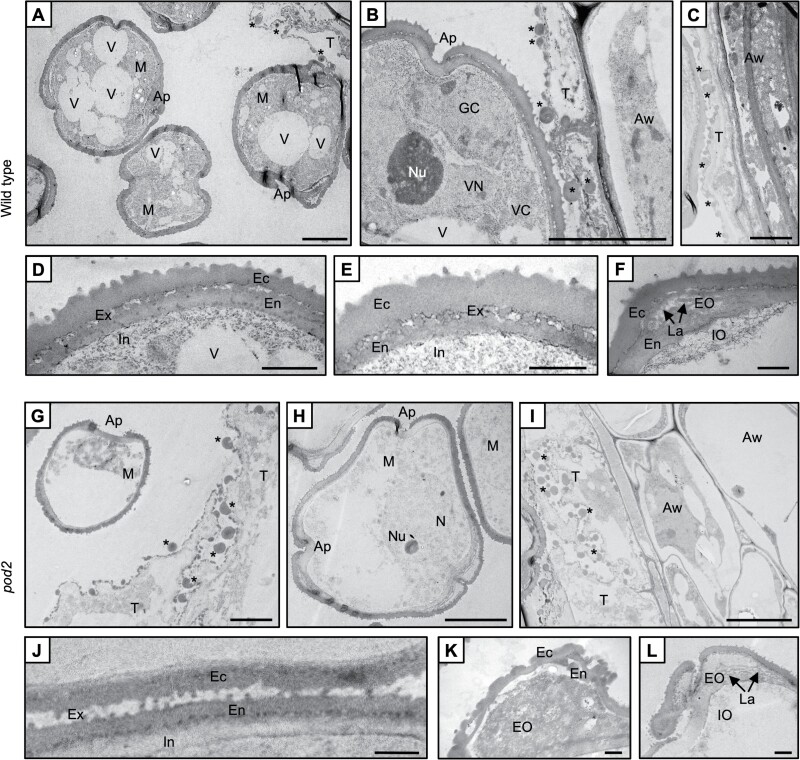
Subcellular features of selected stages of pollen development in wild-type and *pod2* anthers. Cross sections of anthers at the microspore (A, D, G–L), young pollen grain (B), and medium pollen grain (C, E, F) stages. Ap, aperture; Aw, anther wall; Ec, ectexine; En, endexine; EO, exine oncus; Ex, exine; GC, generative cell; In, intine; IO, intine oncus; La, lamella; M, microspores; N, nucleus; Nu, nucleolus; T, tapetum; V, vacuole, VC, vegetative cell cytoplasm; VN, vegetative nucleus; Asterisks, orbicules. Scale bars=5 µm (A, B, G–I), 2 µm (C), and 1 µm (D–F, J–L).

Further analysis of ultrastructural details of the pollen was performed by TEM at selected key stages from microspores to early pollen grains. Wild-type microspores at the early vacuolated stage showed cytoplasmic enrichment in ribosomes, with the prominent presence of large vacuoles ready to fuse into larger ones (Fig. 3A). Tapetal cells began to display signs of degeneration, with the conspicuous presence of large electron-dense orbicules in the tapetal wall facing the anther locule, which became larger and more abundant at the young and medium pollen stages (Fig. 3B, C). At this stage, the tapetum was restricted to a thin layer (Fig. 3C). In addition, both the vegetative cell and the generative cell were clearly differentiated in young pollen grains, with the latter still located close to the pollen wall. The vegetative cell of the young pollen grains presented a cytoplasm filled with numerous ribosomes (Fig. 3B). With regard to the pollen wall, the vacuolated microspore exhibited a recognizable structure with intine and exine (formed by endexine plus ectexine) (Fig. 3D), which became thicker at the early to medium pollen grain stage (Fig. 3E). Apertural regions of the wild-type pollen grains showed canonical structures, particularly from the early pollen stage onwards. Both exine oncus and intine oncus were visible, as well as lamellae taking up part of the exine oncus (Fig. 3F). In the *pod2* mutant, the microspores and vacuolated microspores had a relatively empty cytoplasm, with evident signs of degeneration and only a small number of ribosomes (Fig. 3G, H). No evidence of asymmetrical division of the microspore nucleus or the appearance of a generative cell was observed, and the lack of these features resulted in the absence of sperm cells in the *pod2* mutant. Tapetal cells displayed a lower degree of degeneration and a larger size than those of the wild-type plants (Fig. 3G, I). Although several electron-dense orbicules were observed in the tapetal wall facing the anther locule, most electron-dense globules remained in the internal region of the tapetal cells. Regarding the pollen wall, the *pod2* vacuolated microspore exhibited a recognizable structure with intine and exine (formed by endexine plus ectexine) (Fig. 3J). No additional thickening of the pollen wall was detected. Apertural regions, although presenting some recognizable structures, showed several signs of disorganization, with ectexine and endexine poorly structured and intermingled, a not fully structured exine and intine oncus, and scarce or absent lamellae (Fig. 3K, L).

### *POD2* encodes a G-type lectin receptor kinase

**Fig. 4.**
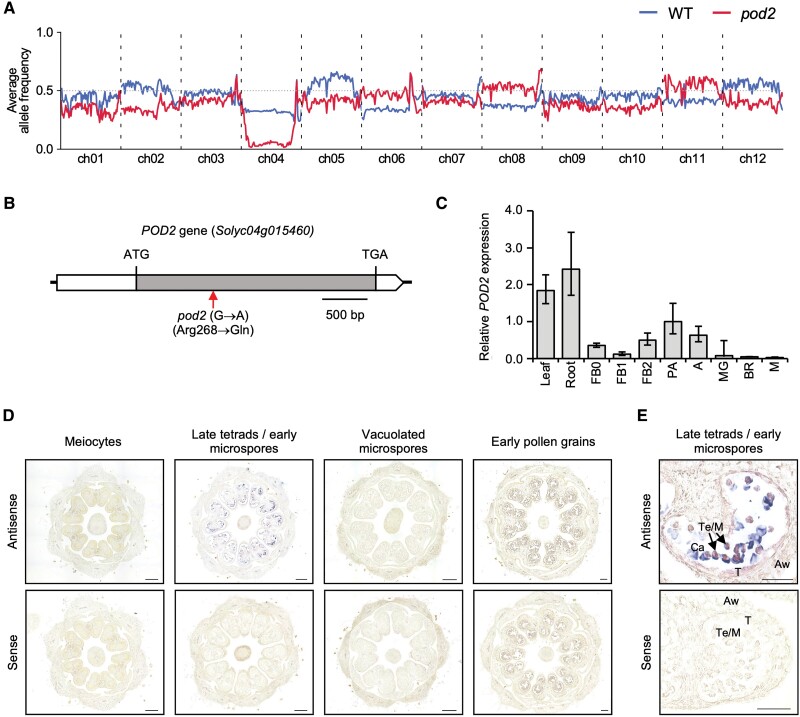
Molecular characterization of the *pod2* mutant. (A) Average allele frequency distribution per chromosome of wild-type (WT; blue line) and *pod2* (red line) pools. (B) Structure and position of the mutation that affects the *POD2* gene in *pod2* mutant plants. The coding sequence is depicted as a grey box. White boxes represent the 5ʹ and 3ʹ untranslated regions. The predicted translation start (ATG) and stop (TAG) codons are indicated. The red arrow indicates the position of the *pod2* point mutation. (C) Relative expression of the *POD2* gene in cv. Moneymaker plants at 10 different stages of plant development. FB0, floral buds of 0–2 mm; FB1, floral buds of 3–4 mm; FB2; floral buds of 4–5 mm; PA, flowers at pre-anthesis stage; A; flowers at anthesis stage; MG; fruits at mature green stage, BR; fruits at breaker stage; M, fruits at mature stage. (D) *In situ* hybridization analysis of *POD2* expression in wild-type entire anthers from meiotic to mitotic developmental stages. (E) *In situ* hybridization analysis of *POD2* expression in wild-type entire anthers at the late tetrad stage. Transverse sections were hybridized with antisense or sense digoxygenin-labeled probe of the *POD2* gene. Aw, anther wall; Ca, callose; M, microspores; T, tapetum; Te, tetrads. Black arrows indicate microspores at the late tetrad stage. Scale bars=200 µm (D) and 50 µm (E).

Genetic analysis performed on a T_1_ segregating population indicated that the *pod2* mutant phenotype was inherited as a monogenic recessive trait (3:1 ratio, 55 wild type:15 *pod2* mutant; χ^2^=0.48; *P*=0.49). Nevertheless, we observed that the *pod2* mutant phenotype did not cosegregate with the T-DNA insertion, suggesting that either somaclonal variation produced during the *in vitro* culture process or a footprint left by an abortive T-DNA insertion could be the underlying cause of the mutant phenotype. Hence, we sought to identify the *pod2* mutation by a mapping-by-sequencing approach, combining bulk segregant analysis with high-throughput sequencing. For that purpose, an F_2_ mapping population was generated by crossing a *pod2* mutant plant to the LA1589 accession of *S. pimpinellifolium*. A total of 117 F_2_ plants were evaluated, of which 23 produced non-viable pollen and small parthenocarpic fruits. The observed segregation ratio (94 wild type:23 *pod2* mutant) in this interspecific F_2_ progeny was consistent with the monogenic recessive inheritance of the *pod2* mutation (χ^2^=1.78, *P*=0.18). To perform mapping-by-sequencing, 25 and 16 F_2_ plants exhibiting wild-type and *pod2* phenotypes, respectively, were selected, and their DNA was extracted, pooled, and sequenced. Genome-wide analysis of the parental allele frequencies in DNA pools revealed a 30 Mb region in chromosome 4 with a strong bias toward *S. lycopersicum* reference alleles (Fig. 4A). An analysis of sequence variants in the region likely to contain the *POD2* gene led to the identification of a point mutation affecting the coding region of the *Solyc04g015460* gene, which encodes the *S. lycopersicum* G-type lectin receptor kinase II.9 (SlG-LecRK-II.9) (Teixeira *et al.*, 2018). The G→A mutation that we identified was predicted to cause the substitution of arginine 268 of the SlG-LecRK-II.9 protein by a glutamine (Fig. 4B).

To examine the spatiotemporal expression patterns of the *Solyc04g015460* gene, RT–qPCR amplifications were performed on RNA extracted from different tissues and developmental stages of wild-type (cv. Moneymaker) tomato plants. The highest expression levels were found in leaves and roots, and the lowest in tomato mature and breaker stages (Fig. 4C). Considering only the reproductive phase, the highest expression levels were found in flowers at the pre-anthesis stage (Fig. 4C). *In situ* hybridization analyses further revealed that *Solyc04g015460* is specifically expressed in anthers (Fig. 4D). Indeed, *Solyc04g015460* gene expression was not detected in the stages of pollen development prior to the tetrad stage; its transcripts started to accumulate in the tapetal cells and microspores at the late tetrad stage when the microspores are released from the callose envelope (Fig. 4E), coinciding with the anther developmental stage at which phenotypic defects began to be observed in *pod2*. Remarkably, *Solyc04g015460* transcripts totally disappeared from the vacuolated microspore pollen stage onwards, indicating that *Solyc04g015460* expression is temporally and spatially stage specific during pollen development. Hence, the observed expression profile pinpoints *Solyc04g015460* as a plausible candidate gene for the *POD2* locus.

**Fig. 5.**
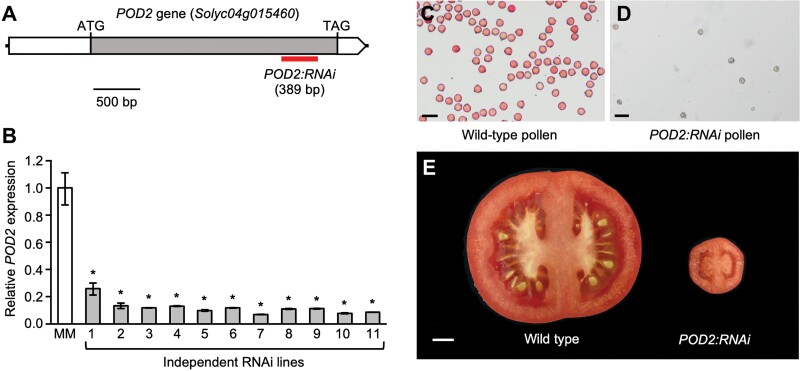
*POD2* gene silencing by RNAi. (A) Position of the sequence targeted by *POD2:RNAi* (indicated by the red box). The coding sequence is depicted as a grey box. White boxes represent the 5ʹ and 3ʹ untranslated regions. The predicted translation start (ATG) and stop (TAG) codons are indicated. (B) Quantitative RT–PCR analysis of *POD2* expression in wild-type cv. Moneymaker (MM) plants and 11 independent *POD2:RNAi* lines. Asterisks indicate significant differences between MM and RNAi plants (*P≤0.001; Student’s *t*-test). (C, D) Pollen of wild-type (C) and *POD2:RNAi* (D) plants stained with TTC. (E) Cross section of wild-type and *POD2:RNAi* tomato fruits. Scale bars=50 µm (C, D) and 1 cm (E).

To confirm that the G→A point mutation in *Solyc04g015460* caused the *pod2* mutant phenotype, we obtained an RNAi construct (*POD2:RNAi*) to silence the expression of this gene, which was then transferred to wild-type cv. Moneymaker plants (Fig. 5A). Afterwards, we used RT–qPCR to assess the extent of *Solyc04g015460* silencing in 11 independent RNAi lines with a similar phenotype to the *pod2* mutant, all of which showed a strong reduction of *Solyc04g015460* mRNA levels compared with wild-type plants (Fig. 5B). Transformant plants carrying the *POD2:RNAi* transgene also phenocopied the *pod2* mutant plants, exhibiting flowers with non-viable pollen (Fig. 5C, D) and small parthenocarpic fruits (Fig. 5E). Taken together, these results indicated that the loss of function of *Solyc04g015460* (hereafter referred to as *POD2*) is responsible for the development of non-viable pollen grains and the production of small parthenocarpic fruits observed in *pod2* mutant plants.

By computational analysis, 73 G-LecRKs have been previously reported in tomato, including POD2, identified as SlG-LecRK-II.9 (Teixeira *et al.*, 2018). G-type LecRLKs contain an α-mannose binding bulb lectin domain, an S-locus glycoprotein domain (SLG), a Plasminogen/Apple/Nematode (PAN) domain, and/or an Epidermal Growth Factor (EGF) domain between the extracellular lectin domain and the transmembrane region (Tang *et al.*, 2017). In particularly, POD2 has an α-mannose binding bulb lectin domain and a PAN domain besides the catalytic domain of the serine/threonine kinases but lacks the SLG and EGF domains (Supplementary Figs S5, S6). To determine whether POD2 was conserved among plants, we carried out an alignment between POD2 orthologs from the major angiosperm lineages (Myburg *et al.*, 2014). High conservation was found among the three domains, but not in the amino acid changed by the *pod2* mutation, which is not located within any putative domain (Supplementary Fig. S5) but is conserved among *Solanaceae* POD2 orthologs (Supplementary Fig. S6). The *pod2* mutation should cause the replacement of an arginine (a positively charged amino acid) by a glutamine (a non-charged residue), suggesting that the specific presence of an arginine or of a positively charged amino acid in this position is relevant for its function. This hypothesis is supported by the fact that *pod2* and the RNAi-silenced plants showed the same phenotype, indicating that *pod2* may also be a null or extremely hypomorphic allele.

### Gene expression changes associated with the loss of *POD2* activity

**Fig. 6.**
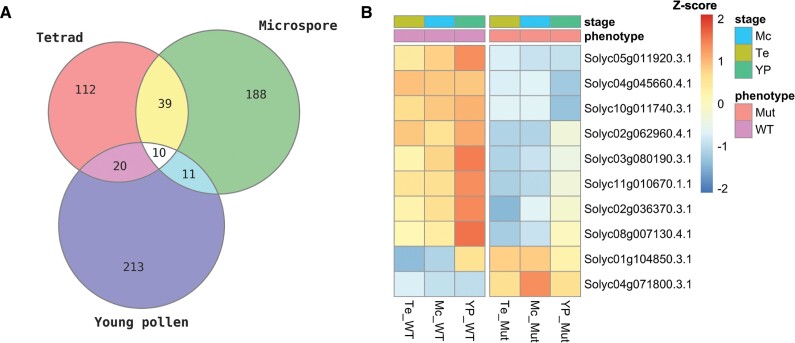
Differentially expressed genes in *pod2* plants. (A) Venn diagrams showing the overlap between differentially expressed genes across three pollen developmental stages. (B) Heatmap of the expression levels of the 10 differentially expressed genes between the wild type (WT) and *pod2* (Mut) in all developmental stages evaluated (Te, tetrad; Mc, microspore; YP, young pollen). The heatmap color scale ranges from blue (low expression) to red (high expression).

To further understand the role of *POD2* in pollen development, we carried out a transcriptomic analysis of wild-type and *pod2* floral buds. Three different pollen developmental stages were evaluated, namely tetrad, microspore, and young pollen, which include the stages where phenotypic differences between the wild type and *pod2* began to be observed in histological studies. We found 181, 248, and 254 genes that were differentially expressed between wild-type and *pod2* floral buds in the tetrad, microspore, and young pollen stages, respectively (Supplementary Dataset S1). Strikingly, only 10 genes were found to be de-regulated at all three developmental stages of *pod2* floral buds, eight of which were down-regulated and two of which were up-regulated (Fig. 6). The up-regulated genes were *Solyc01g104850* and *Solyc04g071800*, encoding a carboxypeptidase and a cytochrome P450 (CYP92B3), respectively. The down-regulated genes were *Solyc04g045660* (encoding the DNA mismatch repair protein MLH1), *Solyc05g011920* (an ACT domain-containing protein), *Solyc10g011740* (a ubiquitin-conjugating enzyme), *Solyc02g062960* (the homeobox-leucine zipper protein HOX14), *Solyc03g080190* (a flavanone 3-hydroxylase-like protein, also named *downy mildew resistance 6-1*; *SlDMR6-1*), *Solyc11g010670* (a 2-oxoglutarate and Fe II dependent dioxygenase), *Solyc02g036370* (a REVEILLE 7-like protein), and *Solyc08g007130* (a beta-amylase). The most up- and down-regulated genes encoded the serine carboxypeptidase and MLH1, respectively.

**Fig. 7.**
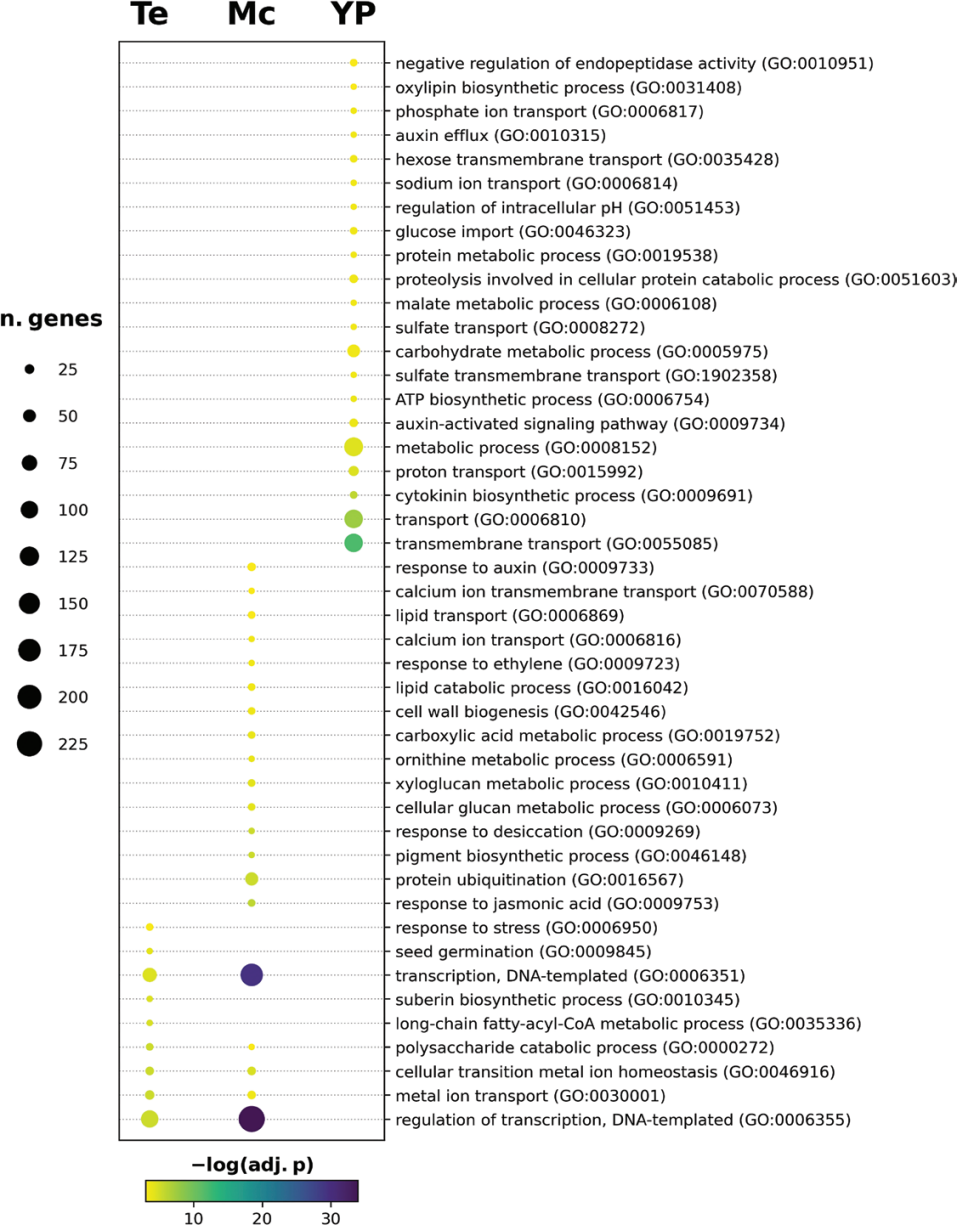
Significantly enriched GO terms in the Biological Process category for differentially expressed genes across developmental stages (Te, tetrad; Mc, microspore; YP, young pollen). The size of the circles indicates the number of genes in the given GO term. Enriched GO terms were determined by FDR adjusted *P*-value (adj.p) ≤0.05. FDR is represented as the –log (adj.p), where the darkest blue are the most significant GO terms. The full list of significantly enriched GO terms is available in Supplementary Dataset S2.

In order to identify de-regulated biological processes in *pod2*, we assessed the enrichment in GO terms (FDR adjusted *P*-value ≤0.05) of differentially expressed genes in the three pollen developmental stages of *pod2* compared with wild-type plants. The most enriched terms included regulation of transcription in the tetrad and microspore stages, and transmembrane transport in the young pollen stage (Fig. 7; Supplementary Dataset S2). Among the de-regulated genes related to transcription regulation, transcription factors belonging to a wide range of gene families were found (Supplementary Fig. S7). The *Solyc02g036370* and *Solyc02g062960* genes, belonging to the MYB and homeobox-leucine zipper families, respectively, stand out for being de-regulated in the three pollen stages studied. In the tetrad and young pollen stages, but not in the microspore stage, we found two down-regulated genes in *pod2* floral buds that encode members of the bHLH (*Solyc03g116340*, which encodes SlbHLH025) and MYB (*Solyc04g005100*) families. In the tetrad and microspore stages, but not in young pollen, five down-regulated genes in *pod2* floral buds were found, which encode a C-repeat binding factor 1 (*Solyc03g026280*), three ethylene response factors (ERF3, *Solyc10g009110*; ERF1a, *Solyc03g093540*; and ERF5; *Solyc03g093560*), and the DNA-binding protein Pti4 (*Solyc05g052050*). At the young pollen stage, all transmembrane transport-related genes studied were de-regulated, whereas none were de-regulated at the tetrad stage and only *Solyc04g070970* (a G-family ABC transporter) was also de-regulated at the microspore stage, showing higher levels of expression in *pod2* floral buds (Supplementary Fig. S8). These results suggest that *POD2* is mainly involved in the control of transcriptional regulation and transmembrane transport mechanisms, which agrees with its predicted role as a transmembrane receptor protein (Teixeira *et al.*, 2018).

## Discussion

In this work, we report the isolation and functional analysis of a novel tomato male-sterile mutant, *pod2*, defective in pollen development, which in turn leads to the formation of small parthenocarpic fruits (Fig. 1A, B). Since Crane (1915) described the first male-sterile tomato mutant, more than 50 male-sterile mutants have been documented by the Tomato Genetics Resource Center (https://tgrc.ucdavis.edu/) and classified into five categories according to the stage at which pollen development aborts: premeiotic, meiotic, tetrad, microspore, and not determined (Gorman and McCormick, 1997). Some of these mutants have been of great interest in agriculture because they have been used or tested for potential application in hybrid seed production, as is the case for *ms-10*, *ms-15*, *ms-32*, and *ms-33* (Gorman and McCormick, 1997; Atanassova, 2000; Sawhney, 2004). Hence, these spontaneous male-sterile mutants offer an excellent experimental system to provide a molecular understanding of pollen development contributing to the employment of male sterility in hybrid seed production, which reduces hybrid seed costs and ensures high varietal purity (Kim and Zhang, 2018). Here, we applied a combined strategy of mapping-by-sequencing and RNAi-mediated gene silencing to reveal that the *pod2* phenotype is caused by a G→A point mutation affecting the *Solyc04g015460* coding region (Figs 4A, B, 5). This gene encodes the G-type lectin receptor kinase SlG-LecRK-II.9, which is expressed in a wide variety of tissues, with root, leaf, and pre-anthesis flower tissues showing the highest level of expression (Fig. 4C). Nevertheless, developmental defects were not detected in *pod2* leaves and roots. Considering that the tomato G-LecRK family includes 73 members (Teixeira *et al.*, 2018), a plausible reason explaining the absence of alterations in *pod2* leaves and roots could lie in the fact that the loss of function of *POD2* may be compensated for by other functionally redundant genes in other tissues and organs, whose expression allows normal plant development and growth. In agreement with this hypothesis, a non-specific pollen expression pattern has also been found for the Arabidopsis L-type lectin receptor kinase *LECRK-IV.2* (*AT3G53810*), whose disruption gives rise to a mutant phenotype that is evident only during pollen development and leads to male sterility (Wan *et al.*, 2008). The function of the closest Arabidopsis homologous gene to *POD2* (*AT5G35370*) has not been described so far, although it was found to be down-regulated in a global expression analysis performed under nematode infection conditions, suggesting a role in the biotic stress response (Fuller *et al.*, 2007). In tomato, phenotypic characterization of the *pod2* mutant and *POD2:RNAi* lines suggested that the function of the SlG-LecRK-II.9 protein is exclusively required for pollen development. The role of *POD2* in male gametophyte formation is reinforced by the fact that it is specifically expressed in tapetal cells and microspores at the late tetrad stage (Fig. 4D, E). Taken together, our results demonstrate that the loss of *POD2* activity leads to the formation of non-viable pollen, resulting in male sterility; they therefore establish an essential role for *POD2/SlG-LecRK-II.9* in the proper development and maturation of pollen grains in tomato.

Pollen ontogeny analysis performed on the *pod2* mutant revealed that it produces non-viable pollen grains due to abnormalities in both gametophytic and sporophytic tissues. The epidermis, endothecium, and middle layers developed without conspicuous alterations. However, developmental differences in the timing and completion of tapetum degeneration were found between wild-type and *pod2* anthers, as the tapetum layer in *pod2* was thicker than the wild-type tapetum from the microspore stage onwards (Fig. 2G–L; Supplementary Fig. S4). Thus, the *pod2* mutation seems to lead to a delay in the induction of tapetum programmed cell death that might impair the optimal formation of pollen grains, as a crucial step during microgametogenesis is the timely coordinated degradation of the tapetum. Indeed, morphological anomalies in *pod2* were first detected at the final stage of microsporogenesis. MMCs were properly formed and underwent meiotic division to form tetrads of haploid microspores encased in callose. However, these microspores and the callose envelope displayed an irregular surface and a distorted appearance (Fig. 2C, F). However, despite these differences, callose envelope degradation was observed and *pod2* haploid microspores were released into the anther locule. Once released, large morphological differences were visible in *pod2* microspores at the microgametogenesis phase (Fig. 2G–L), suggesting that vacuolization, cell wall formation, storage compound accumulation, and mitotic division processes were disrupted by the *pod2* mutation. Thus, histological observations suggest that although MMC meiosis occurs in *pod2*, it does not occur properly, as haploid microspores display anomalies leading to an aberrant process of microgametogenesis. Moreover, ultrastructural analysis confirmed that cytoplasm development, asymmetrical division of microspore nucleus, and full and organized development of the pollen wall and apertures does not occur in *pod2* microspores (Fig. 3). In addition, ultrastructural features of *pod2* tapetal cells differ from those described for wild-type genotypes (Polowick and Sawhney, 1993).

Although *POD2* does not encode a transcription factor, our findings revealed significant alterations in the gene expression profiles of the *pod2* developing anthers, likely caused by an indirect effect associated with the loss of *POD2* activity. These gene expression changes were in agreement with histological observations, as RNA-seq results supported the existence of alterations in MMC meiosis. Thus, *Solyc04g045660*, which encodes a homolog of the DNA mismatch repair protein MLH1, was found to be down-regulated at all three developmental stages of *pod2* floral buds (Fig. 6B). MLH1 proteins are highly conserved and participate in prokaryotic and eukaryotic post-replicative DNA mismatch repair (MMR) pathways fixing the misincorporation of nucleotides and DNA-slippage errors that occur during DNA replication. Homologous chromosome recombination during meiosis involves genome-wide DNA breaks and repairs, in which components of DNA repair pathways, including MMR, also participate. Therefore, MLH eukaryotic proteins, including MLH1 homologs, act in both MMR and meiotic recombination (Pannafino and Alani, 2021). Thus, the loss of function of *MLH1* homologs impairs gamete viability in yeast, animals, and plants. MLH1 depletion in the yeast *Saccharomyces cerevisiae* decreases spore viability (Hoffmann *et al.*, 2003); in zebrafish it causes arrest in spermatogenesis at metaphase I and male sterility (Feitsma *et al.*, 2007); and in Arabidopsis, it results in reduced male and female fertility and defects in pollen grain development (Dion *et al.*, 2007). In tomato, RNAi silencing of other genes encoding components of the MMR pathway, such as *MutS HOMOLOG1*, causes a pleiotropic phenotype including dwarfism and male sterility (Yang *et al.*, 2015). Furthermore, among the down-regulated genes in *pod2* microspores we found *Solyc06g005170*, which is homologous to the Arabidopsis *AtMPK4* gene. Loss of function of *AtMPK4* leads to the failure of MMCs to form a normal intersporal callose wall, preventing the completion of meiotic cytokinesis (Zeng *et al.*, 2011). Likewise, *Solyc08g013940*, the homolog of the Arabidopsis *AtRUNKEL* gene, was up-regulated at the *pod2* tetrad stage. Mutations in *AtRUNKEL* have been found to cause cytokinesis defects resulting in abnormal phragmoplast organization, cell plate expansion arrest, abnormal pollen formation, and reduced male fertility (Krupnova *et al.*, 2013). Therefore, both histological and transcriptomic results point to a functional role of *POD2* in the suitable completion of MMC meiosis, participating in the signaling cascade that orchestrates DNA repair and cytokinesis.

It is also noted that the pollen-specific gene *LATE ANTHER TOMATO 52*, encoding a heat-stable glycosylated protein, was found to be down-regulated in *pod2* at the young pollen stage; this gene has previously been shown to play a key role in pollen hydration and pollen germination in tomato (Muschietti *et al.*, 1994). Furthermore, tomato homologs of other pollen and tapetum development-related genes in Arabidopsis and rice were de-regulated in *pod2* anthers. Among them, the homolog of the Arabidopsis *LAP5/POLYKETIDE SYNTHASE B* gene (*Solyc05g053550*) was down-regulated in *pod2* at the tetrad stage. This gene is specifically expressed in tapetal cells during microspore development in Arabidopsis anthers and is required for the biosynthesis of sporopollenin, the major constituent of exine in the outer pollen wall (Kim *et al.*, 2010). Likewise, the homolog of the rice *CYP703A3* gene (*Solyc05g047680*), a cytochrome P450 hydroxylase, was down-regulated in *pod2* at the microspore stage. Mutations in *CYP703A3* result in defective pollen exine and anther epicuticular layer formation, in contrast to the Arabidopsis *cyp703a2* mutations, which lead only to defective pollen exine (Yang *et al.*, 2014). Additionally, the *Solyc08g008370* gene, encoding a protein with a development and cell death (DCD) domain, was found to be down-regulated in *pod2* at the tetrad and microspore stages. This domain is also found in the rice *DEFECTIVE TAPETUM CELL DEATH 1* (*DTC1*) gene, which controls tapetum degeneration by modulating the dynamics of reactive oxygen species during male reproduction. Indeed, *dtc1* mutants display an enlarged tapetum layer with delayed degeneration, causing male sterility (Yi *et al.*, 2016). The homolog of the Arabidopsis *VANGUARD1* gene (*Solyc03g123620*) was also down-regulated in *pod2* at the microspore stage. *VANGUARD1* encodes a pectin methylesterase, which shows tapetum-specific expression at stages 10–11 of Arabidopsis anther development where the tapetum is undergoing programmed cell death, suggesting that *VANGUARD1* could be involved in modifying material released from the degenerating tapetum to form part of the pollen coat (Phan *et al.*, 2011). Taken together, the transcriptomic data indicated that the loss of *POD2* function would prevent proper tapetum degradation and haploid microspore formation, affecting the subsequent microgametogenesis process and finally leading to the production of non-viable pollen grains.

In addition to playing a crucial role in regulating plant development (Julca *et al.*, 2021), RKs, together with transcription factors, have been revealed to be essential for pollen biogenesis and function on the basis of results from a recent comprehensive expression atlas capturing pollen development in a wide range of plants (Kim *et al.*, 2021). Indeed, RK signaling cascades have been involved in different processes of pollen development, such as the specification of tapetum and microsporocyte identity or the regulation of carbohydrate distribution and utilization (Cai and Zhang, 2018). Our findings support the crucial role of RK proteins in tomato pollen development. Furthermore, the loss of function of the *Solyc04g077010* gene, encoding an LRR-RK protein, impairs male fertility, leading to the formation of parthenocarpic fruits (Takei *et al.*, 2019). Hence, the elucidation of RK function, as well as the characterization of kinase domain target proteins and ligands, would result in valuable insight into the contribution of RK proteins to the regulation of tomato pollen development. These findings would additionally contribute to a deeper understanding of early fruit development mechanisms, including flower-to-fruit transition in the absence of pollination, which results in the development of seedless parthenocarpic fruits, a valued trait in tomato breeding programs for quality and processing attributes, as well as in hybrid production.

## Supplementary data

The following supplementary data are available at *JXB* online.

Fig. S1. Hierarchical clustering of common expressed genes.

Fig. S2. Phenotype of the *pod2* mutant.

Fig. S3. Pollen viability in a hand-pollination control test.

Fig. S4. Comparison of tapetal thickness in wild-type and *pod2* plants.

Fig. S5. Sequence conservation among plant POD2 orthologs.

Fig. S6. Sequence conservation between *Solanaceae* POD2 proteins.

Fig. S7. Heatmap of differentially expressed genes associated with transcription GO terms.

Fig. S8. Heatmap of differentially expressed genes associated with transport GO terms.

Table S1. Oligonucleotide sequences.

Dataset S1. List of significantly differentially expressed genes for *pod2* floral buds in the tetrad, microspore, and young pollen stages of pollen development.

Dataset S2. List of significantly enriched GO terms for the Biological Process category in differentially expressed genes in *pod2* floral buds in the tetrad, microspore, and young pollen stages of pollen development.

erac419_suppl_Supplementary_Dataset_S1

erac419_suppl_Supplementary_Dataset_S2

erac419_suppl_Supplementary_Material

## Data Availability

The DNA-seq and RNA-seq data were deposited at the Sequence Read Archive (https://www.ncbi.nlm.nih.gov/sra/) under BioProject accession numbers PRJNA760263 and PRJNA760265, respectively. All other data supporting the findings of this study are available within the paper and within its supplementary data published online.

## Abbreviations

FDR: false discovery rate
G-LecRK: G-type lectin receptor kinase
GO: Gene Ontology
LecRK: lectin receptor kinase
LM: light microscopy
LRR-RK: leucine-rich repeat receptor kinase
MMC: microspore mother cell
MMR: DNA mismatch repair
*ms*: *male sterile*
*pod2*: *pollen deficient 2*
RK: receptor kinase
RNAi: RNA interference
SEM: scanning electron microscopy
SlG-LecRK-II.9: *Solanum lycopersicum* G-type lectin receptor kinase II.9
TEM: transmission electron microscopy
TPM: transcript per million
TTC: 2,3,5-triphenyltetrazolium chloride
