# Supplementary material for: Tomato POLLEN DEFICIENT 2 encodes a G-type lectin receptor kinase required for viable pollen grain formation
Source: J Exp Bot. 2022 Oct 19;74(1):178–93. doi: 10.1093/jxb/erac419 (PMC9786849; doi:10.1093/jxb/erac419)
Supplement: erac419_suppl_Supplementary_Material [file erac419_suppl_supplementary_material.pdf]

The tomato *POD2* encodes a G-type lectin receptor kinase required for viable pollen grain formation

Micol-Ponce, Rosa[1,†,#] (rmicol@ual.es), García-Alcázar, Manuel[1,#] (mga800@ual.es), Lebrón, Ricardo[1] (rlebron@ual.es), Capel, Carmen[1] (ccapel@ual.es), Pineda, Benito[2] (bpineda@btc.upv.es), García-Sogo, Begoña[2] (bgarcia@btc.upv.es), Alché, Juan de Dios[3] (juandedios.alche@eez.csic.es), Ortiz-Atienza, Ana[1] (anaortiz@ual.es), Bretones, Sandra[1] (sba557@ual.es), Yuste-Lisbona, Fernando Juan[1] (fyuste@ual.es), Moreno, Vicente[2] (vmoreno@ibmcp.upv.es), Capel, Juan[1] (jcapel@ual.es), Lozano, Rafael[1,*] (rlozano@ual.es)

[1]Centro de Investigación en Biotecnología Agroalimentaria (CIAIMBITAL). Universidad de Almería. 04120 Almería, Spain.

[2]Instituto de Biología Molecular y Celular de Plantas (UPV-CSIC). Universidad Politécnica de Valencia. 46011 Valencia, Spain.

[3]Departamento de Bioquímica, Biología Celular y Molecular de Plantas, Estación Experimental del Zaidín-CSIC, 18008 Granada, Spain.

[†]Current address: Instituto de Bioingeniería, Universidad Miguel Hernández, Campus de Elche, 03202 Elche, Alicante, Spain.

[#]Rosa Micol-Ponce and Manuel García-Alcázar should be regarded as joint First Authors.

***Corresponding author**

Prof. Rafael Lozano.

Departamento de Biología y Geología (Genética). Edificio CITE II-B, Universidad de Almería. Carretera de Sacramento s/n, 04120 Almería, Spain.

Phone: +34 950 015111. Fax: +34 950 015476. Email: rlozano@ual.es

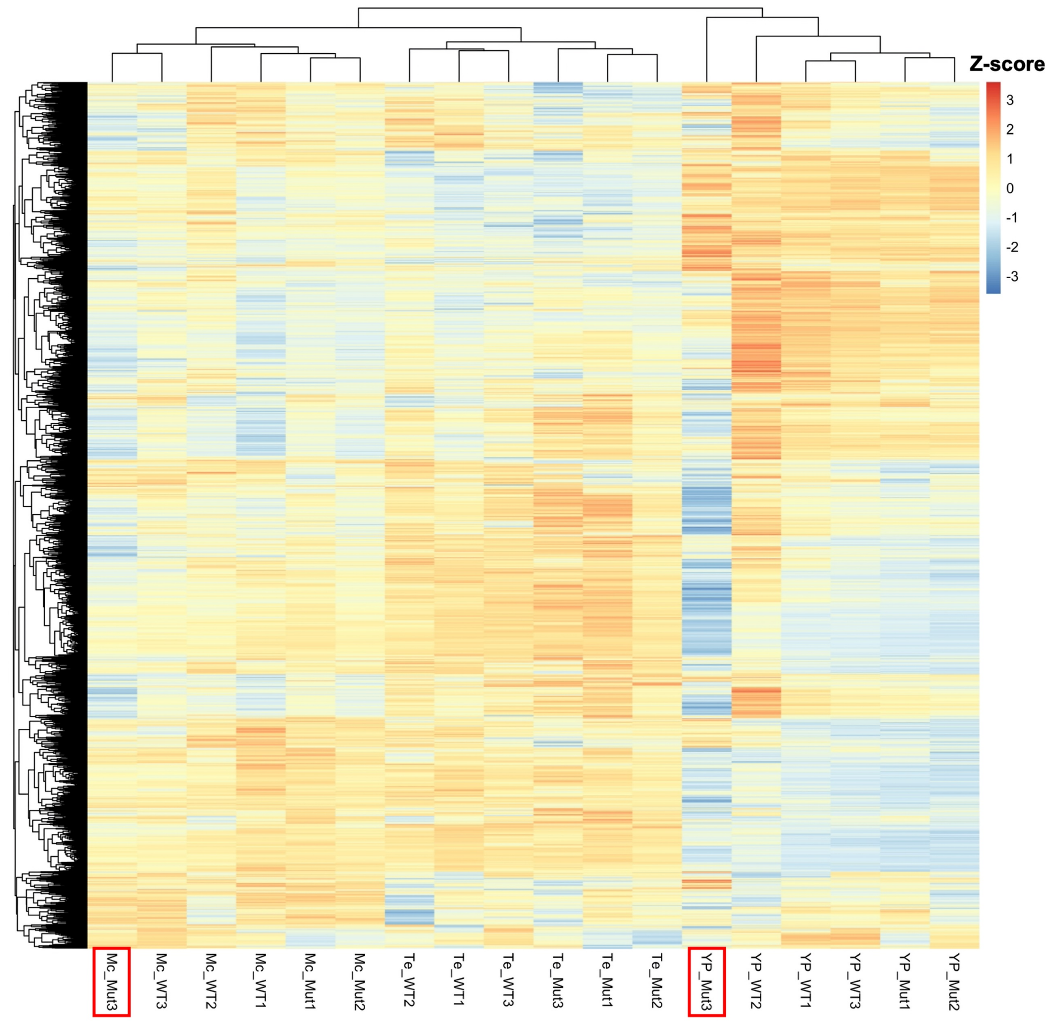

Supplementary Fig. S1. Hierarchical clustering of common expressed genes. The common top-ranked 5000 expressed genes were obtained from all replicates (1, 2 and 3) and developmental stages (Microspore, Mc; Tetrad, Te; Young Pollen, YP) in wild-type (WT) and *pod2* (Mut) plants. The red box indicates the two replicates removed in the RNA-seq analysis.

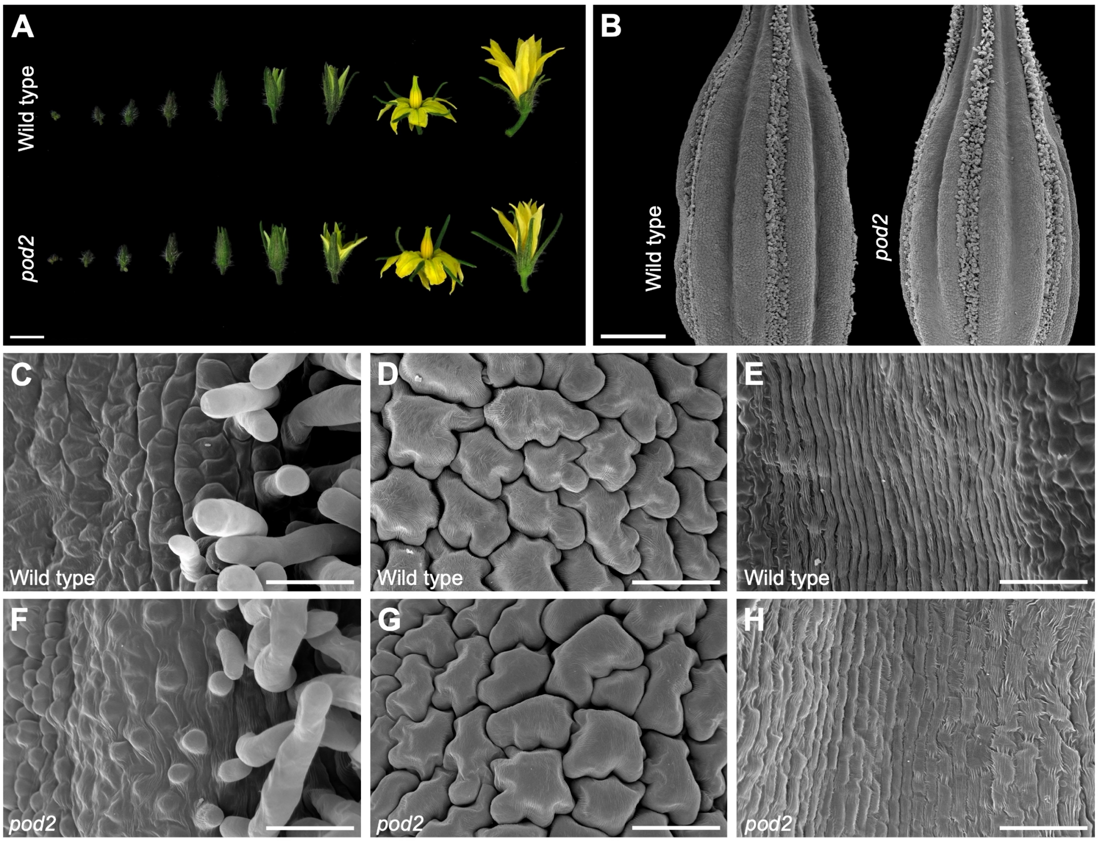

Supplementary Fig. S2. Phenotype of the *pod2* mutant. (A) Floral development in wild-type and *pod2* plants. (B-H) Morphology of epidermal stamen cells observed under Scanning Electron Microscope (SEM). (B) Whole stamen of wild-type and *pod2* plants. Cells from three parts of the stamen: ventral (C, F), dorsal-media (D, G), and dorsal-distal (E, H). Scale bars: 1 cm (A), 1 mm (B), and 50 μm (C-H).

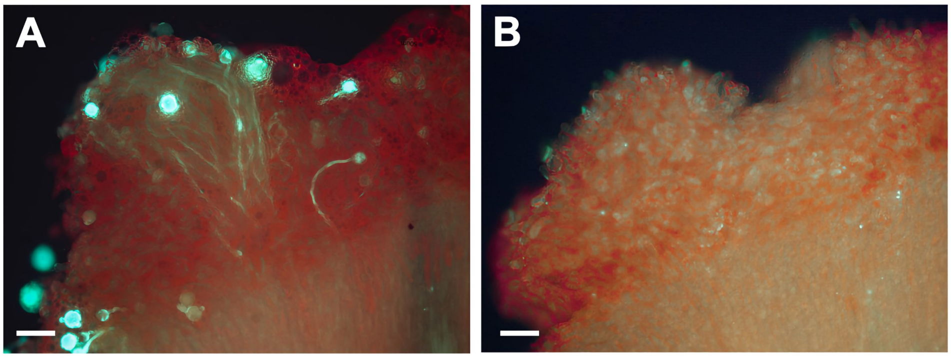

Wild-type pollen in wild-type stigma *pod2* pollen in *pod2* stigma

Supplementary Fig. S3. Pollen viability in hand-pollination control test. (A, B) Hand self-pollination of wild-type pollen in wild-type stigma (A), and *pod2* pollen in *pod2* stigma (B). Photographs were taken under an Optiphot-2 (Nikon) optical microscope equipped with an HB-10101AF Mercury Lamp (Nikon), using aniline blue staining to observe pollen tube development. Scale bars: 100 μm.

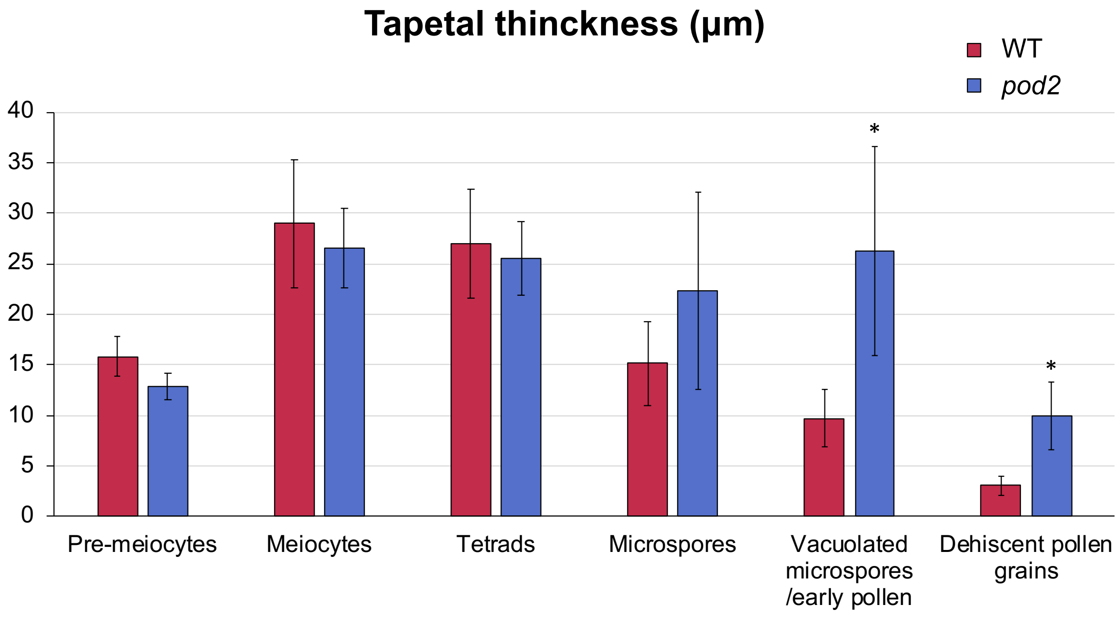

Supplementary Fig. S4. Comparison of tapetal thickness in wild-type (WT) and *pod2* plants. Tapetal thickness was evaluated in anthers at different developmental stages: pre-meiocytes, meiocytes, tetrads, microspores, vacuolated microspores/early pollen, and dehiscent pollen grains. Error bars represent the standard deviation of the mean values. Asterisks indicate significant differences between *pod2* and wild-type plants in a Student's t-test ($P \leq 0.001$).

```
Oryza           ------------------------------------------------------------  0
Phoenix         ------------------------------------------------------------  0
Arabidopsis     ------------------------------------------------------------  0
Vitis           MNCTASNHLLFTTSPNLTLRGDRSPNQLLFFFCKIPTPSPNGNIPQTYHTRQNSVCGRVH  60
Glycine         ------------------------------------------------------------  0
Solanum         ------------------------------------------------------------  0
Populus         ------------------------------------------------------------  0

Oryza           ------------------------------------------------------------  0
Phoenix         ------------------------------------------------------------  0
Arabidopsis     ------------------------------------------------------------  0
Vitis           SPHFCSSQDPIEKKTHVITEIFVLFVFSLTLLCIRLLSNALLPDFAQRWRSLVAFSEEAE  120
Glycine         ------------------------------------------------------------  0
Solanum         ------------------------------------------------------------  0
Populus         ------------------------------------------------------------  0

Oryza           ------------------------------------------------------------  0
Phoenix         ------------------------------------------------------------  0
Arabidopsis     ------------------------------------------------------------  0
Vitis           ARVSAYPSHLWKAIVAYEDRRFFSHFGVDPVGIARAALSLSALGGGSTITQQVIHQINDG  180
Glycine         ------------------------------------------------------------  0
Solanum         ------------------------------------------------------------  0
Populus         ------------------------------------------------------------  0

Oryza           -----------------------------------------------------------M  1
Phoenix         ------------------------------------------------------------  0
Arabidopsis     -----------------------------------------------------------M  1
Vitis           SRLGHQSKSIWCCERECFSSQREPRGRSGQKKWRKPEQTNSKSSSTPLIKPICWFSQFFS  240
Glycine         ------------------------------------------------MSQICTS-----  7
Solanum         ------------------------------------------------------------  0
Populus         ------------------------------------------------------------  0

Oryza           PPSRSLACLLLPPLLLLLESVRDADAGPLAVEIVRPSFTATSYDYVDT-GGAFLVSRNGS  60
Phoenix         --MAT----PFVVVLLLLLYPFTSSAAVVATEFLYPNFTATVINYVDN-AGVFLTSRNAT  53
Arabidopsis     KS--TF---LLLLLLLSLNLLFVFVSCASSIEFVYPNFTASNLRFVDSSKGAFLLSRNSI  56
Vitis           PSMLYM---GFLVFLSTILLSFSLVCGLSSSELIYPNFTASNFNFVEY-NGAFLFSRNET  296
Glycine         -SNYTM---KPIITFSCILLFFTTISARSFSDYITPNFTASYLEFIDN-FGTFLFSHNRT  62
Solanum         --MGSS---FLLFIVTLLLSCFIVHSGPLSLQPLTPNFTASNFKFIDT-SGSFLSSPNGT  54
Populus         --MGSS---FLFLFFSSTLLPYLCISGPSTVQTIKQPFTASHFFFIDQ-SGVFLISSNGN  54
                              .          .     : :   ***:   :::   * ** * *

Oryza           FRAAVFNPGKQQ--ASFYLAVLHAPSGTPVWSANRDAPTSSTGKVQLSVGGITVSDA---  115
Phoenix         FKAAIYNAGQQP--SHYYLTLLHGPTSTPVWSANRNSPIPNDGLVILSPVGLSVVHP---  108
Arabidopsis     FKAGLFSPGGDDSSTGFYFSVVHVDSGSTIWSSNRDSPVSSSGTMNLTPQGISVIEDG--  114
Vitis           FKVAMFNPGAQQ--KNFYLCIIHVASGAVIWSANRDAPVSNYGKMNLTINGITVTDQG--  352
Glycine         FKAAIFNPGGQQ--TSFYLCVIHAASNTIIWSGNRDAPISDSGKMLLSFKGITILDEH--  118
Solanum         FKAAITNTKPQE--RSYYFVIVHSESHVVVWSANRDMPVSDSGELRLSVDGLTLFD----  108
Populus         FTASISNSE-EN--PPYYFCITHVKSNAIIWIANRNHPISDSDKLYLTTNGLAINSTYNS  111
                * ..: .   :     :*: : *  :   :* .**: *  . . : *:  *:::

Oryza           -NGTVLWSTPPL--RSPVAALRLQDTGDLQLLDAGNATLWRSFDNATDTLLPGQQLLAGA  172
Phoenix         -NGSLLWSTPPL--PSPVRALRLLDTGNLLLVNASNASLWRSFDHPTDTLLSDQPLRAGS  165
Arabidopsis     KSQIPVWSTPVL--ASPVKSLRLTDAGNLLLLDHLNVSLWESFDFPTDSIVLGQRLKLGM  172
Vitis           GS--VKWGTPPL--KSSVSALLLAETGNLILLDQFNGSLWQSFDYPTDTIVIGQRLSVGT  408
Glycine         GN--TKWSTPSL--KSQVNRLQLTEMGNLVLLDKSNGSLWESFQNPTDTIVIGQRLPVGA  174
Solanum         DSGDTVWSAKRSSTSSSVTSMQLLESGNLVLVDAFNKSVWESFDSPTDTIVVGQRLPVGK  168
Populus         STTSVVWSTEGLSPSSQVSAMELRDSGNLVLLNRNNVSLWESFDQPTDTIVMGQSLAVGT  171
                 .    *.:      * *  : * : *:* *::  * ::*.**:  **::: .* *  *
```

```
Oryza        YLSSAKGATDFSQGDYRFGVITADVLLTWQG-STYWRLSNDARGFKDTNAAVASMSVNAS   231
Phoenix      SLTSPVSDTNFSEGDYRLTVTTSDAVMTWKESQQYYSISTDARSFKDDNADISFMVSNST   225
Arabidopsis  FLSGSVSRSDFSTGDYKFLVGESDGLMQWRG-QNYWKLRMHIRANVDSNFPVEYLTVTTS   231
Vitis        SLSGALSDNDLSTSDYRFVVSTSNAIMQWHG-LTYWKLSMDSSAYKNSNYLVEYMAMNQT   467
Glycine      SLSSAASNSDLSKGNYKLTITSSDAVLQWYG-QTYWKLSTDTRVYKNSNDMLEYMAINNT   233
Solanum      SLVSSVKEDELAKGDYELVVVENDAMLQWNE-KTYWKLSMEPKAFTDAYTPVEYMMISSN   227
Populus      SVDCYNAENDMSVGDYRLVVTGGDAVLQWNG-MSYWKLSMEPKGSQDSKVPVSFLALNDT   230
              :         ::: .:*.: :    : :: *     *: :  .       :    :  :  . .

Oryza        GLFAVAADGAMVFRVG----LAPAEFRMLKLGSDGRLRIISYALVNSSAPVGGDFIAPAG   287
Phoenix      GLYLLSTNQKVIFHVF----LSASSFRIMKLDSAGRFQIISYSAANSSSSLDDKFVAPSS   281
Arabidopsis  GLALMARNGTVVVVRVALP--PSSDFRVAKMDSSGKFIVSRFSGK----NLVTEFSGPMD   285
Vitis        GLFLFGRNGSVVVIQMDLS--P-SDFRIAKLDASGQFIISTLSGT----VLKQEYVGPKD   520
Glycine      GFYLFGDGGTVF--QLGLP--L-ANFRIAKLGTSGQFIVNSFSGTN---NLKQEFVGPED   285
Solanum      GLFLLGANGTDRVIQVNLDELKDPDFRIAKLEENGHFSVKRLSNG----NWMSEFDSPID   283
Populus      GLFLLGSDRSTVVIKLT---LGPADFRVAKLGFDGKLSVRKFVDQ----NWVQEFVSPAD   283
             *:   .. .                .**: *:    *:: :                .:  .* .

Oryza        DCDLPLQCPSLGYCSPA--GNGSTCTCPPLFAASVTVAGSCTPGDGSTLASPAACQNNDS   345
Phoenix      DCDLPFSCQTLGLCNTG--ANGTSCTCPVLFAAS--KTGGCSPADDSL-L-ASTCGANF-   334
Arabidopsis  SCQIPFVCGKLGLCNLDNASENQSCSCPDEMRMDAG-KGVCVPVSQSL-SLPVSCEARN-   342
Vitis        ACRIPFICGRLGLCTDDTASNSPVCSCPSGFRADPKSVTNCVPSDSSY-SLPSPCNLTNS   579
Glycine      GCQTPLACGRAGLCTENTVSSSPVCSCPPNFHVGSGTFGGCEPSNGSY-SLPLACKNSSA   344
Solanum      SCRVAFTCKKLGVCDE------GRCSCPPGFRVSSEVNGSCAPVDRNL-VMPVSCNASLN   336
Populus      ECQIPLSCNKMGLCSS------GRCSCPPNFHGDPLSKSGCTPTDASL-ALPSGCSNGK-   335
              *    : *    * *         *:**  :  .        * *  . .        *

Oryza        ---SSGGASVSYIALKPLTSYFATKFDAPTNTGVNKTACRALCTASCACLGFFHDSVSLS   402
Phoenix      --------SISYLSPGSGIVYFANKFMVPITVGQGISACQSLCSGNCSCVAFIYRNSSRS   386
Arabidopsis  ---------ISYLELGLGVSYFSTHFTDPVEHGLPLLACHDICSKNCSCLGVFYENTSRS   393
Vitis        VSQSNLS-VVSYLMLAFGVEYFANNFWEPVQYGVNLSVCENLCSGDCSCLGIFHENSSGS   638
Glycine      FSFLNIG----------YVEYFGNFYSDPVLYKVNLSACQSLCSSNCSCLGIFYKSTSGS   394
Solanum      MNVTELGNRVSYLRLENGLDYFANDFIEPVKRGVNVSACQDLCSKNCSCLSVFHDQSSGS   396
Populus      ----ELNSSVFYVNLGSELDYFANGFMAPAKRDINLLACQDLCTRNCSCLGIFYGNSSGS   391
                                   **.. :  *          .*. :*: .*:*:..:: . * *

Oryza        CRLIGGKQLGSLYKGAS----DTNLGYIKTFNSATKAGSN-------QIGSSSANHTVPI   451
Phoenix      CYLIE-NQLGSVFNSKDGE--TASSIYVKTLLSGSP-QPI--------SGKSTSRHLIPI   434
Arabidopsis  CYLVK-DSFGSLSLVKNSPENHDLIGYVKLSIRKTNAQPP----GNNNRGGSSFPVIALV   448
Vitis        CYLVE-NVLGSLISSST-N-ENVQLGYIKVLVGSS---PNMDGNNSSSNQSQEFPIAALV   692
Glycine      CYMIE-NELGSIQSSNG-GDERDILGFIKAITVASTTSSNDGNDDKENSQNGEFPVAVAV   452
Solanum      CYMIE-NFLGSILRGSDSGNGRGRLGYVKVISEPSLFDPN----DNSSDKRSRLPVVALV   451
Populus      CYLIE-NPLGSIMEASSSN--SKRLGYVKTIVVSS----R----ANKVNESAKFPIVGLV   440
             * ::   .  :**:                 ::*       :                  :

Oryza        VLPSVAAFLL--LAVLGWYIWW-RNKMS-KNGRKKKGKSS-TMKVYLGRQKSPSRDTG--   504
Phoenix      LLPTIAAFLLIIVVIFMGILWWTRQQQRKRGPSIRRSKSAVMKEIQLGRQKSRTMVATAG   494
Arabidopsis  LLPCSGFFLL----IALGLLWW-RRCAVMRYSS--------IREKQVTR-----------   484
Vitis        LLPSTGFFLF----VALGFLWW-RRWGFSKN-----------RDLKLGH-----------   725
Glycine      LLPIIGFIIL----MALIFLVW-RRLTLMSK----------MQEVKLG------------   485
Solanum      LLPSSGLFLI--IVMMAGIMWLMRRKRLMQI-----------SGKEIRR-----------   487
Populus      LLPSSGILLI--IIVVLGFICW-RRNRLYRT-----------AKLKLGR-----------   475
             :**   . :::     :     :    *.                    :

Oryza        --YNADADDDGGGDDDDIVIPGMPARFSYQEITTMTSNFATKVGSGGFGTVYKGELPGGE   562
Phoenix      DKGRSSGNHESDDDDDDISILGLPTRFTYAELEAATWNFRTKIGSGGFGAVYKGELPD-E   553
Arabidopsis  ------PGSFESGDLGSFHIPGLPQKFEFEELEQATENFKMQIGSGGFGSVYKGTLPD-E   537
Vitis        ------SSSPSSDDLDAFSIPGLPIRFEYEEIEAATDNFKTQIGSGGFGAVYKGIMPD-K   778
Glycine      ------KNSPSSGDLDAFYIPGLPARFDYEELEEATENFKTLIGSGGFGTVYKGVLPD-K   538
Solanum      ------TDSSSSADLDSISILGLPVKFDHEEIRVATECFRNQIGTGGFGTVYKGTLSD-G   540
Populus      ------G-DSSSSELEIISIPGLPVRFNYEDLVAATESFSTQIGSGGFGTVYKGTLPD-K   527
                            . :   : *  *:*  :*  .  ::    *    *   :*:****:****  :  .
```

```
Oryza          GLIAVKKLEAAGVQAKREFCTEITIIGNIRHVNLVRLRGFCAEGSRRLLVYEYMNRGSLD   622
Phoenix        TLVAVKKIDNVGLQGKKEFCTEIAVIGNIRHVNLVRLRGFCAEGTKRLLVYEYMSRGSLD   613
Arabidopsis    TLIAVKKITNHGLHGRQEFCTEIAIIGNIRHTNLVKLRGFCARGRQLLLVYEYMNHGSLE   597
Vitis          TLVAVKKITNLGVQGKKEFCTEIAVIGNIHHVNLVKLKGFCAKGRQRLLVYEYMNRGSLD   838
Glycine        SVVAVKKIGNIGIQGKKDFCTEIAVIGNIHHVNLVKLKGFCAQGRHRLLVYEYMNRGSLD   598
Solanum        AVVAVKKMNALGAHGNREFCTEIAIIGRVHHVNLVSLKGFCAHRGERFLVYEYMNRGSLD   600
Populus        SVVAVKKITNVGVQGKKEFCTEIAIIGSTRHVNLVKLKGFCAQGRQRFLVYEYMNRGSLD   587
                ::****:   * :..::*****::**  :*.*** *:****.  . :******.:***:

Oryza          RSLFGRTGPVLEWGERMEVAIGAARGLAYLHTGCEQKIVHCDVKPENILLANGGQVKISD   682
Phoenix        RSLFG-VGPVLEWQERIDIAIGAARGLAYLHTGCEHKIVHCDVKPENILLDERGQARIGD   672
Arabidopsis    KTLFSGNGPVLEWQERFDIALGTARGLAYLHSGCDQKIIHCDVKPENILLHDHFQPKISD   657
Vitis          RTLFS-NGPVLEWQERVDIALGTARGLAYLHSGCEHKIIHCDVKPENILLHDNFQAKISD   897
Glycine        RNLFG-GEPVLEWQERFDVALGTARGLAYLHSGCVQKIIHCDIKPENILLQDQFQAKISD   657
Solanum        RTLFG-HGPALDWHTRYEIALGTARGLAYLHGGCEQKIIHCDVKPENILLHDNLQVKISD   659
Populus        RTLFG-NGPVLKWQERFEIALGTARGLAYLHSYCERKIIHCDVKPENILLHDNLQVKISD   646
               :.**.   *.*.*  * ::*:*:********  * :**:***:******* :  * :*.*

Oryza          FGLAKLMSREQSALFTTMRGTRGYLAPEWISNAAISDRADVYSFGMVLLELIHGRKNRGE   742
Phoenix        FGLAKLLRPEQSGLFTTMRGTRGYLAPEWLTNSAISDWTDVYSFGMVLLELVRGRKNKSV   732
Arabidopsis    FGLSKLLNQEESSLFTTMRGTRGYLAPEWITNAAISEKADVYSYGMVLLELVSGRKNCSF   717
Vitis          FGLSKLLSPEESSLFTTMRGTRGYLAPEWLTSSAISDKTDVYSFGMVLLELVSGRKNCSL   957
Glycine        FGLSKLLSAEQSGLFTTMRGTRGYLAPEWLTNSAITEKTDVYSFGMVLLELVSGRKNCYY   717
Solanum        FGLSKLLNSEQSSWFTTMRGTRGYLAPEWLTSSAITEKSDVYSYGMVLLEIVRGKKNSSF   719
Populus        FGLSKLLTPEQSSLFTTMRGTRGYLAPEWLAGVTISDKADVYSYGMVLLEIVRGRKNSAA   706
               ***:**:  *:*. ***************::. :*:: :****:******:: *:**

Oryza          QEAAAPANNVAVAAGSGEHSDLPSGWSSAMTSTASGTSGGGDEYFPMLALELHEQRRYLD   802
Phoenix        VPSEGESE----SAGCSE--------------STGSGGGGGGRYFPMVALEAHEQGRYGE   774
Arabidopsis    RSRSNSVT----EDNNQN-------------HSSTTTTSTGLVYFPLYALDMHEQGRYME   760
Vitis          RTQSHSTD----DGGSGG-------------GHSPLSSGPEPVYFPLFALEMHEQGRYLE   1000
Glycine        RSRSHSMD----DSNSGG-------------GNSSTSSTTGLVYFPLFALEMHEQRSYLE   760
Solanum        QPPNDTTS----QSESSEMN---------RLSPSSLASANQSIYFPLFALEMHEQKKYLE   766
Populus        QPQSRSIE-----NDSSEGN---------GTSSSSSGWEPRSAYFPLHALEMHEKKRYSE   752
                                                :         ***: **: **:  * :

Oryza          LVDARLEGRVDEAEAARTVRVALCCLHEDPALRPSMATVVRILEGSVPPPEPRVEALGFL   862
Phoenix        LADPRLEGRAPAAEVERVVRVALCCLHEEPGLRPAMGSVVAMLERTAEVGEPRVESLNFL   834
Arabidopsis    LADPRLEGRVTSQEAEKLVRIALCCVHEEPALRPTMAAVVGMFEGSIPLGNPRMESLNFL   820
Vitis          LADPRLEGRVASEEVEKLVLVALCCVHEEPTLRPCMVSVVGMLEGGITLSQPRTESLNFL   1060
Glycine        LADSRLEGRVTCEEVEKLVRIALCCAHEEPALRPNMVTVVGMLEGGTPLPHPRIESLNFL   820
Solanum        LVDPRVLGNVKSEEVEKLVRVALCCLHEEPTLRPTMANVVGMLEGVFPLATPQVQSLNFL   826
Populus        LADSRLERRVANEEVEKLVKVALCCLHEDPTLRPTMVNVVGMLEGITPLAEPRQESLNFL   812
               *.* *:  ..   *. : * :**** **:* *** *  ** ::*       *: ::*.**

Oryza          RLYGRSYPLPVPGSLTAMAGGGSHLD---------------------ESLKDTSAPR   898
Phoenix        RLYGRGFAEPAGNASL--FGTGGHNI----GSVCSPATATSSGSPSYLSSQQVSGPR   885
Arabidopsis    RFYGLRFAESSMVEGQ--NGESETMVFHRRES---SNSGGSRQSASYIASQEVSGPR   872
Vitis          RFYGRRFTEASMVEET--DGQQTVVLYPQANA-YLTSISGHTSFSYISSQQISGPR   1114
Glycine        RFYGRRYTEASTIAEE--NEYGSVMLQQARSSTTSMPSDSSTRGFSYMSSQNISGPR   875
Solanum        RFYGRRFTEASMIGGD--QEVNVFELHQQNRN-ISSTTSSSYNSFSYMSSQQVSGPR   880
Populus        RFYGRRFSEASRIEGS--NERNEFGLFPQA-N-LTSGTSSSYTSMSYLSAQQLSGPR   865
               *:**  :                                         : :: *.**
```

Supplementary Fig. S5. Sequence conservation among plant POD2 orthologues. Multiple amino acid sequence alignment of full-length POD2 orthologues from different angiosperm lineages, as classified in Myburg *et al.* (2014): *Phoenix dactylifera* XP_017699622 (representing Arecales), *Oryza sativa* LOC_Os09g28180 (Poales), *Arabidopsis thaliana* NP_198387 (Malvids), *Populus trichocarpa* XP_024447662 (Malpighiales), *Glycine max* NP_001238617

(Fabids), *Solanum lycopersicum* XP_004237111 (Asterids) and *Vitis vinifera* VW17745 (representing Vitales). Identical and similar residues across all sequences are shaded in black and grey, respectively. Asterisks and periods indicate identical and conserved residues, respectively. Numbers indicate residue positions. The red, green and blue lines highlight the D-mannose binding lectin domain, the PAN/APPLE-like domain and the catalytic domain of the Serine/Threonine kinases, respectively. The predicted amino acid change caused by the *pod2* mutation studied in this work is highlighted in red. This alignment was obtained using Clustal Omega (https://www.ebi.ac.uk/Tools/msa/clustalo/) and shaded with Boxshade 3.21 (http://www.ch.embnet.org/software/BOX_form.html).

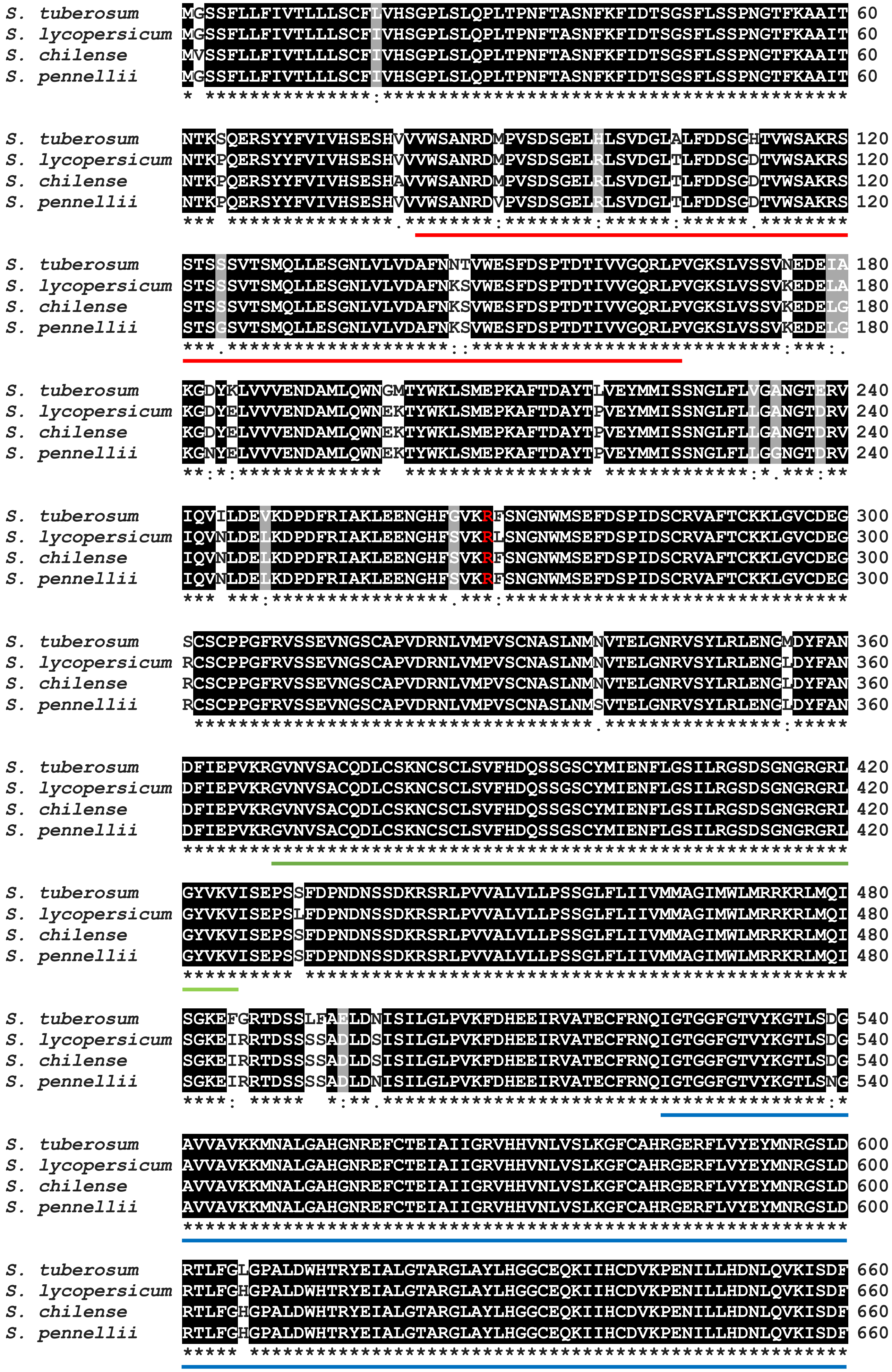
S. tuberosum MGSSFLLFIVTLLLSCFLVHSGPLSLQPLTPNFTASNFKFIDTSGSFLSSPNGTFKAAIT 60
S. lycopersicum MGSSFLLFIVTLLLSCFIVHSGPLSLQPLTPNFTASNFKFIDTSGSFLSSPNGTFKAAIT 60
S. chilense MVSSFLLFIVTLLLSCFIVHSGPLSLQPLTPNFTASNFKFIDTSGSFLSSPNGTFKAAIT 60
S. pennellii MGSSFLLFIVTLLLSCFIVHSGPLSLQPLTPNFTASNFKFIDTSGSFLSSPNGTFKAAIT 60
S. tuberosum NTKSQERSYYFVIVHSESHVVVWSANRDMPVSDSGELHLSVDGLALFDDSGHTVWSAKRS 120
S. lycopersicum NTKPQERSYYFVIVHSESHVVVWSANRDMPVSDSGELRLSVDGLTLFDDSGDTVWSAKRS 120
S. chilense NTKPQERSYYFVIVHSESHAVVWSANRDMPVSDSGELRLSVDGLTLFDDSGDTVWSAKRS 120
S. pennellii NTKPQERSYYFVIVHSESHVVVWSANRDVPVSDSGELRLSVDGLTLFDDSGDTVWSAKRS 120
S. tuberosum STSSSVTSMQLLESGNLVLVDAFNNTVWESFDSPTDTIVVGQRLPVGKSLVSSVNEDEIA 180
S. lycopersicum STSSSVTSMQLLESGNLVLVDAFNKSVWESFDSPTDTIVVGQRLPVGKSLVSSVKEDELA 180
S. chilense STSSSVTSMQLLESGNLVLVDAFNKSVWESFDSPTDTIVVGQRLPVGKSLVSSVKEDELG 180
S. pennellii STSGSVTSMQLLESGNLVLVDAFNKSVWESFDSPTDTIVVGQRLPVGKSLVSSVKEDELG 180
S. tuberosum KGDYKLVVVENDAMLQWNGMTYWKLSMEPKAFTDAYTLVEYMMISSNGLFLVGANGTERV 240
S. lycopersicum KGDYELVVVENDAMLQWNEKTYWKLSMEPKAFTDAYTPVEYMMISSNGLFLLGANGTDRV 240
S. chilense KGDYELVVVENDAMLQWNEKTYWKLSMEPKAFTDAYTPVEYMMISSNGLFLLGANGTDRV 240
S. pennellii KGNYELVVVENDAMLQWNEKTYWKLSMEPKAFTDAYTPVEYMMISSNGLFLLGGNGTDRV 240
S. tuberosum IQVILDEVKDPDFRIAKLEENGHFGVKRFSNGNWMSEFDSPIDSCRVAFTCKKLGVCDEG 300
S. lycopersicum IQVNLDELKDPDFRIAKLEENGHFSVKRLSNGNWMSEFDSPIDSCRVAFTCKKLGVCDEG 300
S. chilense IQVNLDELKDPDFRIAKLEENGHFSVKRFSNGNWMSEFDSPIDSCRVAFTCKKLGVCDEG 300
S. pennellii IQVNLDELKDPDFRIAKLEENGHFSVKRFSNGNWMSEFDSPIDSCRVAFTCKKLGVCDEG 300
S. tuberosum SCSCPPGFRVSSEVNGSCAPVDRNLVMPVSCNASLNMNVTELGNRVSYLRLENGMDYFAN 360
S. lycopersicum RCSCPPGFRVSSEVNGSCAPVDRNLVMPVSCNASLNMNVTELGNRVSYLRLENGLDYFAN 360
S. chilense RCSCPPGFRVSSEVNGSCAPVDRNLVMPVSCNASLNMNVTELGNRVSYLRLENGLDYFAN 360
S. pennellii RCSCPPGFRVSSEVNGSCAPVDRNLVMPVSCNASLNMSVTELGNRVSYLRLENGLDYFAN 360
S. tuberosum DFIEPVKRGVNVSACQDLCSKNCSCLSVFHDQSSGSCYMIENFLGSILRGSDSGNGRGRL 420
S. lycopersicum DFIEPVKRGVNVSACQDLCSKNCSCLSVFHDQSSGSCYMIENFLGSILRGSDSGNGRGRL 420
S. chilense DFIEPVKRGVNVSACQDLCSKNCSCLSVFHDQSSGSCYMIENFLGSILRGSDSGNGRGRL 420
S. pennellii DFIEPVKRGVNVSACQDLCSKNCSCLSVFHDQSSGSCYMIENFLGSILRGSDSGNGRGRL 420
S. tuberosum GYVKVISEPSSFDPNDNSSDKRSRLPVVALVLLPSSGLFLIIVMMAGIMWLMRRKRLMQI 480
S. lycopersicum GYVKVISEPSLFDPNDNSSDKRSRLPVVALVLLPSSGLFLIIVMMAGIMWLMRRKRLMQI 480
S. chilense GYVKVISEPSSFDPNDNSSDKRSRLPVVALVLLPSSGLFLIIVMMAGIMWLMRRKRLMQI 480
S. pennellii GYVKVISEPSSFDPNDNSSDKRSRLPVVALVLLPSSGLFLIIVMMAGIMWLMRRKRLMQI 480
S. tuberosum SGKEFGRTDSSLFAELDNISILGLPVKFDHEEIRVATECFRNQIGTGGFGTVYKGTLSDG 540
S. lycopersicum SGKEIRRTDSSSSADLDSISILGLPVKFDHEEIRVATECFRNQIGTGGFGTVYKGTLSDG 540
S. chilense SGKEIRRTDSSSSADLDSISILGLPVKFDHEEIRVATECFRNQIGTGGFGTVYKGTLSDG 540
S. pennellii SGKEIRRTDSSSSADLDNISILGLPVKFDHEEIRVATECFRNQIGTGGFGTVYKGTLSNG 540
S. tuberosum AVVAVKKMNALGAHGNREFCTEIAIIGRVHHVNLVSLKGFCAHRGERFLVYEYMNRGSLD 600
S. lycopersicum AVVAVKKMNALGAHGNREFCTEIAIIGRVHHVNLVSLKGFCAHRGERFLVYEYMNRGSLD 600
S. chilense AVVAVKKMNALGAHGNREFCTEIAIIGRVHHVNLVSLKGFCAHRGERFLVYEYMNRGSLD 600
S. pennellii AVVAVKKMNALGAHGNREFCTEIAIIGRVHHVNLVSLKGFCAHRGERFLVYEYMNRGSLD 600
S. tuberosum RTLFGLGPALDWHTRYEIALGTARGLAYLHGGCEQKIIHCDVKPENILLHDNLQVKISDF 660
S. lycopersicum RTLFGHGPALDWHTRYEIALGTARGLAYLHGGCEQKIIHCDVKPENILLHDNLQVKISDF 660
S. chilense RTLFGHGPALDWHTRYEIALGTARGLAYLHGGCEQKIIHCDVKPENILLHDNLQVKISDF 660
S. pennellii RTLFGHGPALDWHTRYEIALGTARGLAYLHGGCEQKIIHCDVKPENILLHDNLQVKISDF 660

```
S. tuberosum     GLSKLLNSEQSSWFTTMRGTRGYLAPEWLTSSAITEKSDVYSYGMVLLEIVRGKKNSSFQ 720
S. lycopersicum  GLSKLLNSEQSSWFTTMRGTRGYLAPEWLTSSAITEKSDVYSYGMVLLEIVRGKKNSSFQ 720
S. chilense      GLSKLLNSEQSSWFTTMRGTRGYLAPEWLTSSAITEKSDVYSYGMVLLEIVRGKKNSSFQ 720
S. pennellii     GLSKLLNSEQSSWFTTMRGTRGYLAPEWLTSSAITEKSDVYSYGMVLLEIVRGKKNSSFQ 720
                 ************************************************************

S. tuberosum     PPNDTTSQSESSERNRLSPSSLASANQPIYFPLFALEMHEQKKYLELVDPRVLGSVKSEE 780
S. lycopersicum  PPNDTTSQSESSEMNRLSPSSLASANQSIYFPLFALEMHEQKKYLELVDPRVLGNVKSEE 780
S. chilense      PPNDTTSQSESSEMNRLSPSSLASANQSIYFPLFALEMHEQKKYLELVDPRVLGSVKSEE 780
S. pennellii     PPNDTTSQSESSEMNRLSPSSLASANQSIYFPLFALEMHEQKKYLELVDPRVLGNVKSEE 780
                 ************* ************* **************************.*****

S. tuberosum     VEKLVRVALCCLHEEPTLRPTMANVVGMLEGVLPLATPQVQSLNFLRFYGRRFTEASTID 840
S. lycopersicum  VEKLVRVALCCLHEEPTLRPTMANVVGMLEGVFPLATPQVQSLNFLRFYGRRFTEASMIG 840
S. chilense      VEKLVRVALCCLHEEPTLRPTMANVVGMLEGVFPLATPQVQSLNFLRFYGRRFTEASMIV 840
S. pennellii     VEKLVRVALCCLHEEPTLRPTMANVVGMLEGVFPLATPQVQSLNFLRFYGRRFTEASMIG 840
                 ********************************:************************ * 

S. tuberosum     GDQEVNVFELHQQSRNRSSTTSSSYNSFSYMSSQQVSGPR                     880
S. lycopersicum  GDQEVNVFELHQQNRNISSTTSSSYNSFSYMSSQQVSGPR                     880
S. chilense      GDQEVNVFELHQQNRNLSSTTSSSYNSFSYMSSQQVSGPR                     880
S. pennellii     GDQEVNVFELHQQNRNLSSTTSSSYNSFSYMSSQQVSGPR                     880
                 *************.** ***********************
```

Supplementary Fig. S6. Sequence conservation between Solanaceae POD2 proteins. Sequence alignment of full-length POD2 orthologues from *Solanum lycopersicum* XP_004237111, *Solanum tuberosum* PGSC0003DMT400070026, *Solanum chilense* TMW91996 and *Solanum pennellii* XP_015072266. Other details are as described in the legend of Supplementary Figure S5.

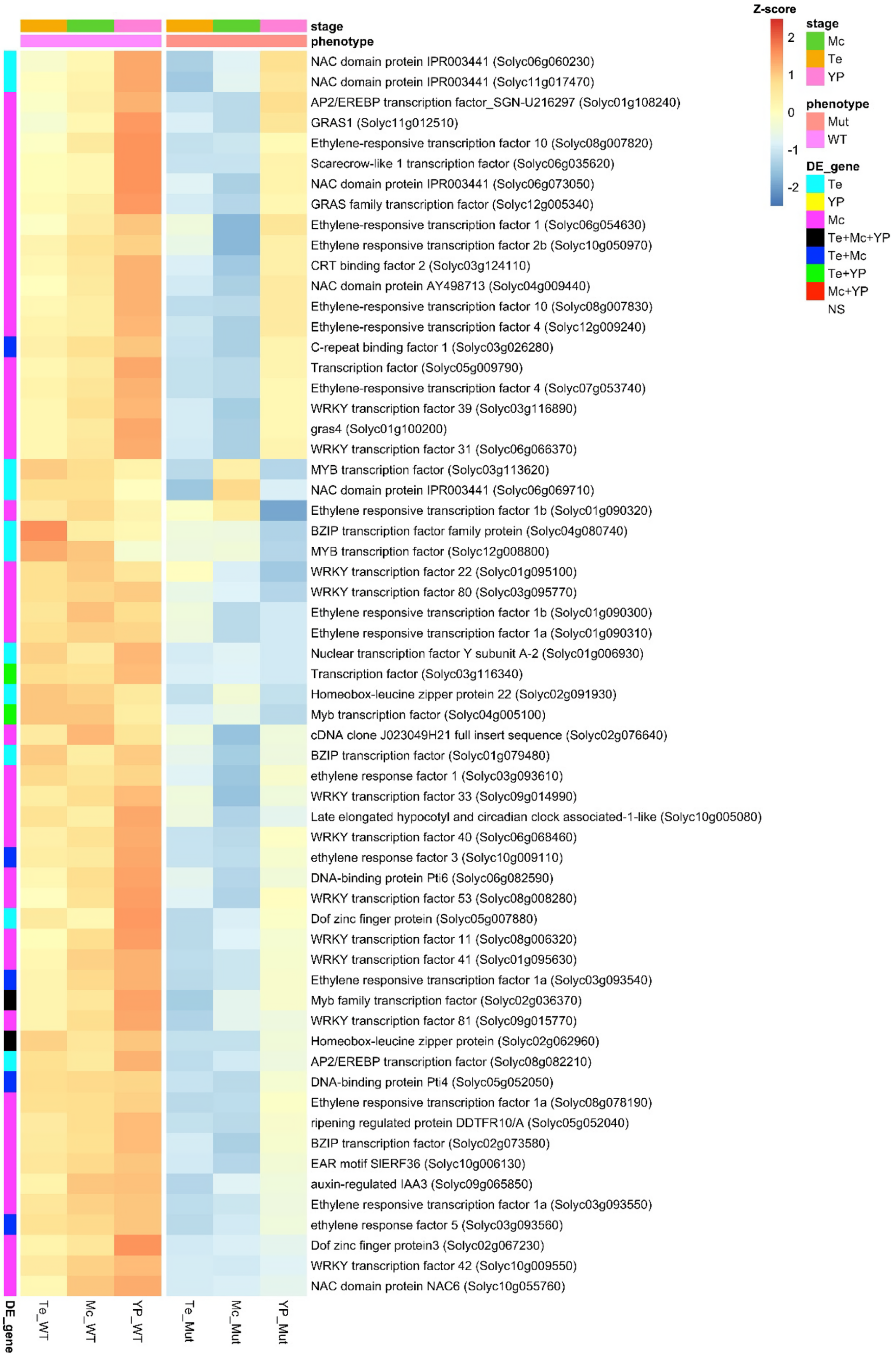

Supplementary Fig. S7. Heatmap of differentially expressed genes associated with transcription GO terms. Heatmap of the expression levels of differentially expressed genes between the wild-type (WT) and *pod2* (Mut) annotated with GO terms involved in transcription (GO:0006355, regulation of transcription, DNA-templated; GO:0006351, transcription, DNA-templated). Te, tetrad; Mc, microspore; YP, young pollen. Heatmap color scale range from blue (low expression) to red (high expression).

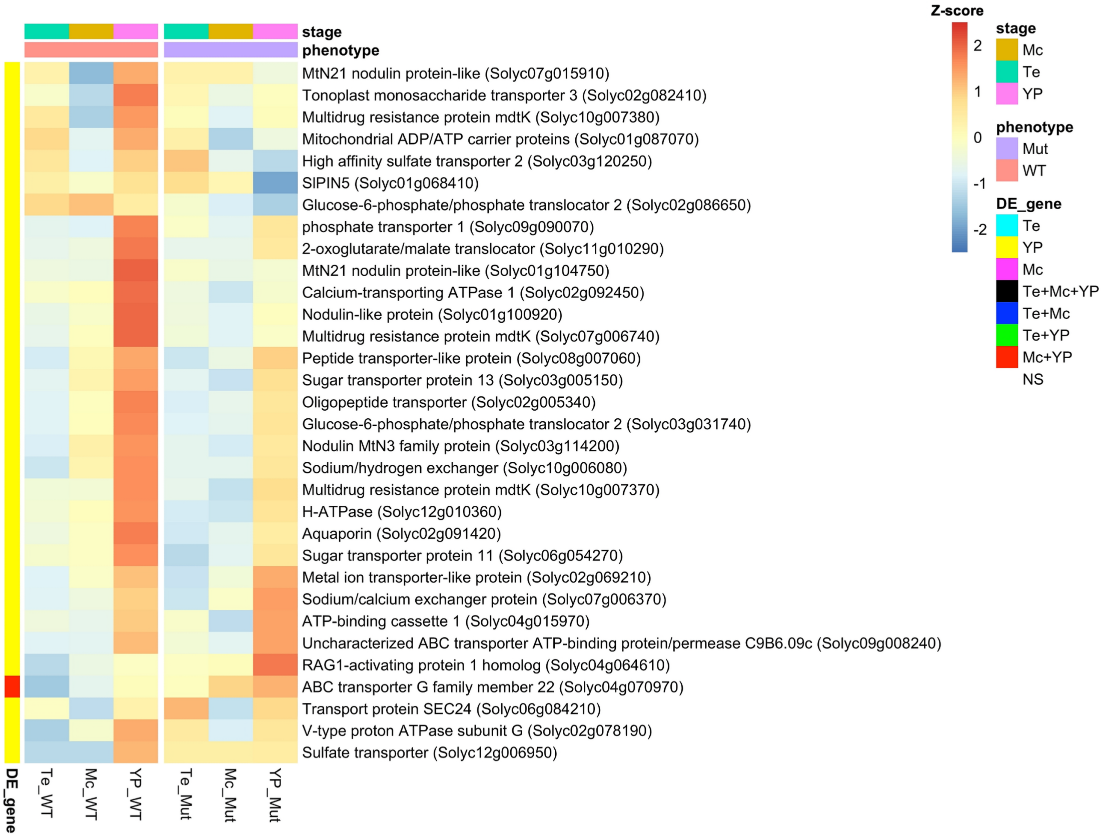

Supplementary Fig. S8. Heatmap of differentially expressed genes associated with transport GO terms. Heatmap of the expression levels of differentially expressed genes between the wild-type (WT) and *pod2* (Mut) annotated with GO terms involved in transport (GO:0055085, transmembrane transport; GO:0006810, transport). Te, tetrad; Mc, microspore, and YP, young pollen. Heatmap color scale range from blue (low expression) to red (high expression).

Supplementary Table S1. Oligonucleotides sequences.

Purpose	Oligonucleotide name(s)	Oligonucleotide sequence (5′→3′)	
		Forward primer (F)	Reverse primer (R)
Sequencing of *POD2* cDNA	POD2-Seq-F1/R1	CCATTGAAGAAGCACAACCA	TGCATACCGTACCAAGTTGC
	POD2-Seq-F2/R2	AGGTAAAGTAGGTTGACTCGCTAC	ATTCGCCCCTAAAAGGAACA
	POD2-F1/R1	TGGAACCAAAGGCTTTTACTG	TGTTCCAAATCCACCAGTACC
	POD2-Seq-F3/R3	GCATTTCCATACTGGGGTTG	AGCCATTCGGGAGCTAAGTA
	POD2-Seq-F4/R4	TATCAGATTTCGGGCTTTCC	CCCTGTGAAAACCAGCAACT
Genotyping of *pod2*	POD2-F1/R1	TGGAACCAAAGGCTTTTACTG	TGTTCCAAATCCACCAGTACC
Construction of *RNAI:POD2*	RNAi_POD2_F/R	CTAGACTCGAGACTTCCCATTATTC GCGTTG	ATCGATGGTACCTGGACCTGAA ACTTGCTGTG
qRT-PCR analysis	UBQ3-F/R	CCAAGATCCAGGACAAGGAA	AAATCAAACGCTGCTGGTCT
	POD2-Fz/Rz	CAAAAATGTTGGCACAGTCG	TGTTCGGACGCTTCAAAAAT
in situ hybridization	POD2-Fis/Ris	GGGGAATGTCAAAAGTGAGG	TAACCTCTTGATCCCCACCG
